# Supplementary material for: Active and dynamic mitochondrial S-depalmitoylation revealed by targeted fluorescent probes
Source: Nat Commun. 2018 Jan 23;9:334. doi: 10.1038/s41467-017-02655-1 (PMC5780395; doi:10.1038/s41467-017-02655-1)
Supplement: Supplementary file 1 — Supplementary Information [file 41467_2017_2655_MOESM1_ESM.pdf]

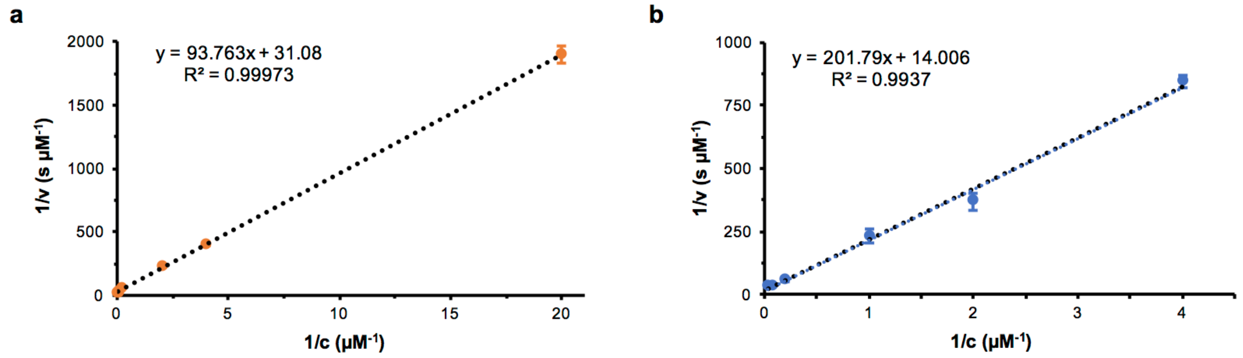

Supplementary Figure 1: Lineweaver-Burk plots of mitoDPP-2 (a) and mitoDPP-3 (b) with 50 nM APT1.

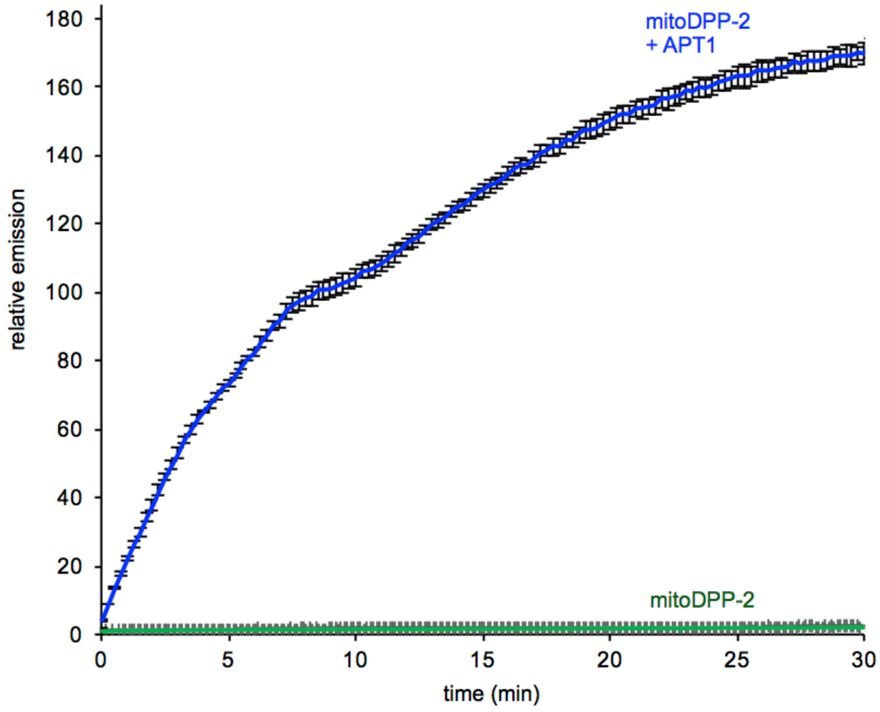

Supplementary Figure 2: Enzymatic Kinetics of mitoDPP-2 with APT1 at higher pH. *In vitro* fluorescence assay of mitoDPP-2 (1 μM) in HEPES (20 mM, 150 mM NaCl and pH 8.0) with 50 nM purified APT1 (λ_{ex} 490/20 nm, λ_{em} 545/20 nm). Error bars are ± s.e.m. (n = 4).

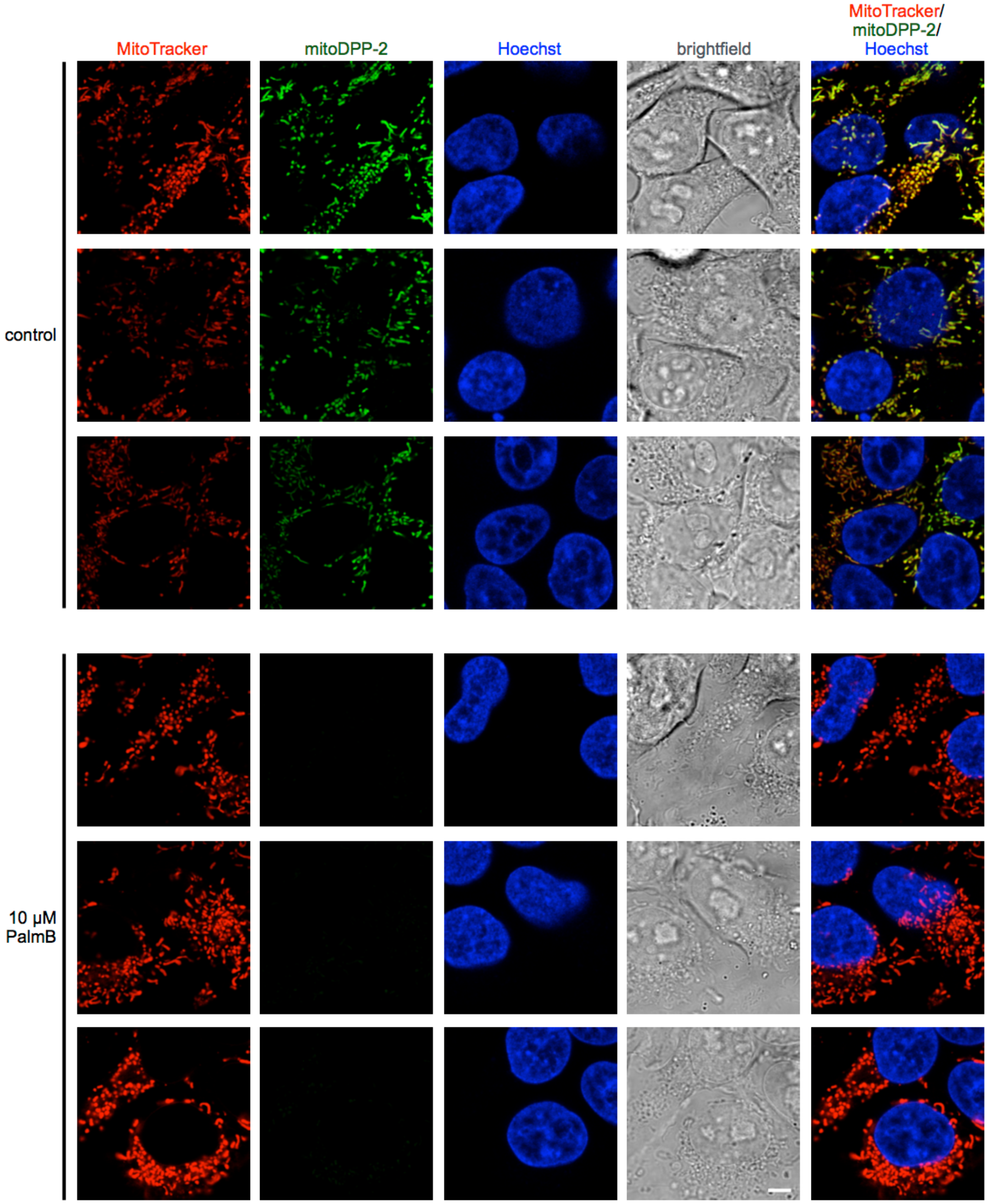

Supplementary Figure 3: Complete imaging series from Fig. 3a. HEK293T cells treated for 30 min with 1 μM Hoechst 33342, 100 nM MitoTracker Deep Red, and either DMSO or 10 μM PalmB, washed, loaded with 250 nM mitoDPP-2 for 10 min, and then analyzed by confocal fluorescence microscopy. Images for MitoTracker, mitoDPP-2, Hoechst 33342 nuclear stain, brightfield, and an overlay of MitoTracker, mitoDPP-2, and Hoechst 33342 are shown for each set of conditions. Three representative images are shown for each condition. 5 μm scale bar shown.

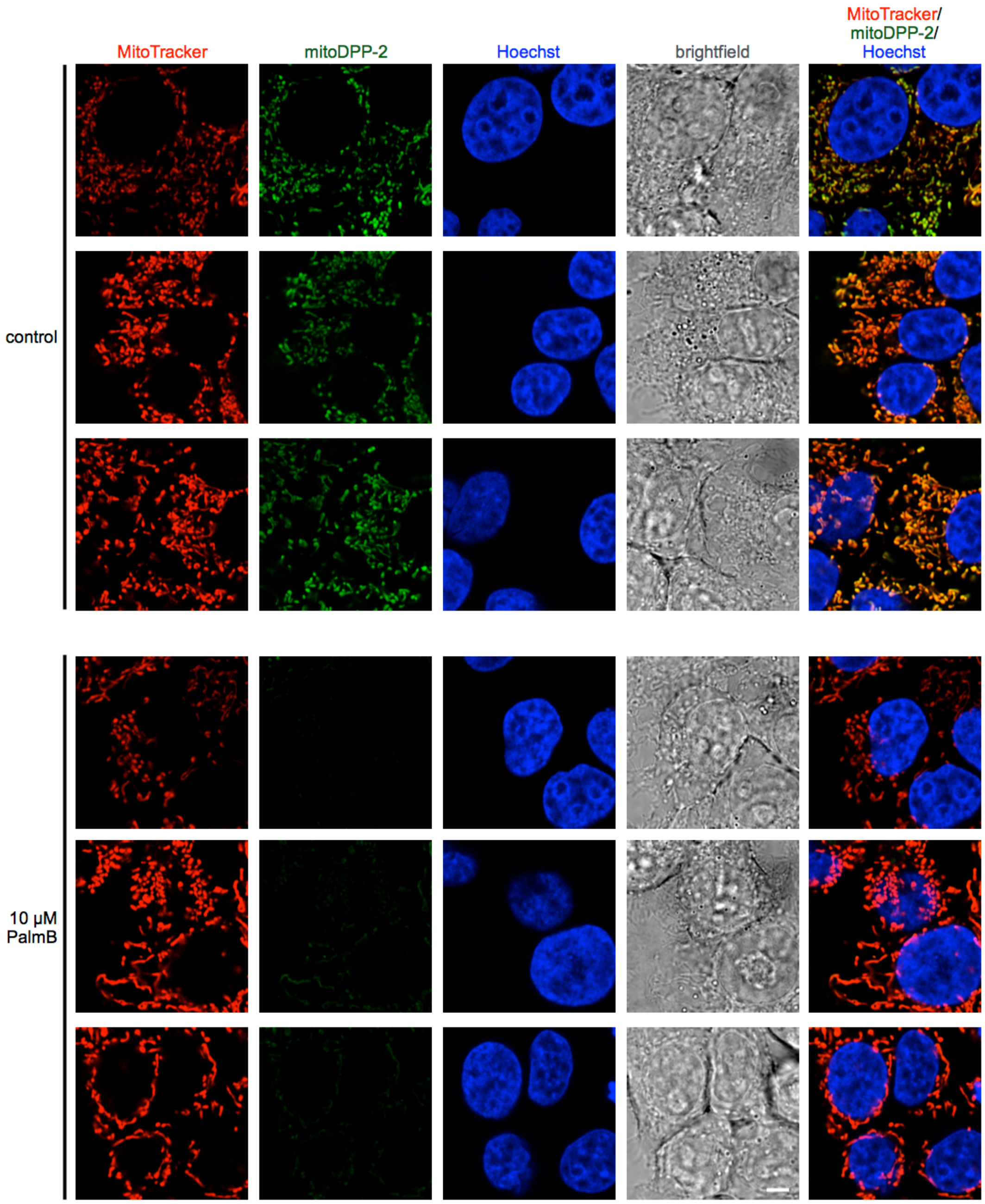

Supplementary Figure 4: Complete imaging series from Fig. 3d. MCF-7 cells treated for 30 min with 1 μM Hoechst 33342, 100 nM MitoTracker Deep Red, and either DMSO or 10 μM PalmB, washed, loaded with 250 nM mitoDPP-2 for 10 min, and then analyzed by confocal fluorescence microscopy. Images for MitoTracker, mitoDPP-2, Hoechst 33342 nuclear stain, brightfield, and an overlay of MitoTracker, mitoDPP-2, and Hoechst 33342 are shown for each set of conditions. Three representative images are shown for each condition. 5 μm scale bar shown.

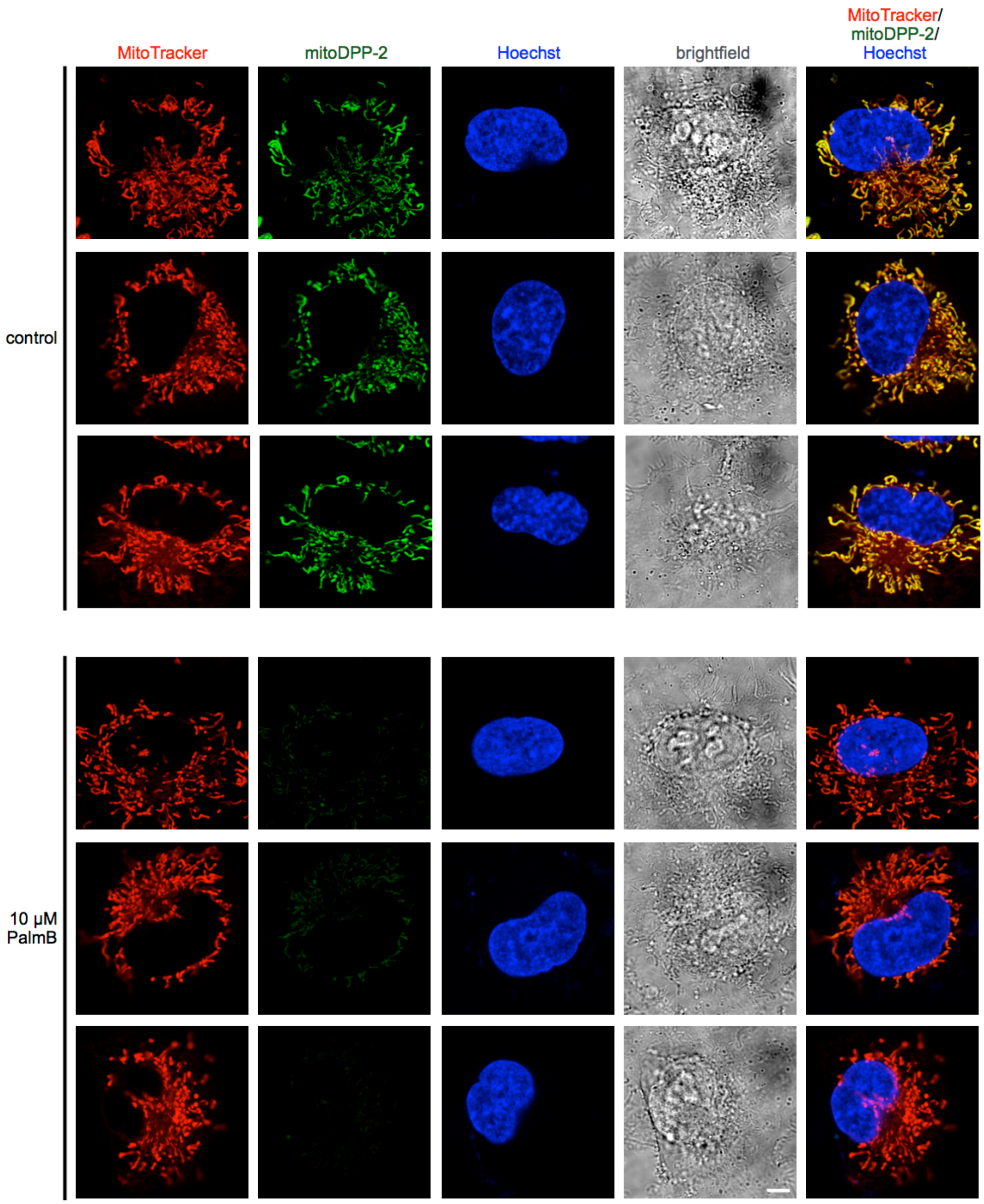

Supplementary Figure 5: Complete imaging series from Fig. 3g. A549 cells treated for 30 min with 1 μM Hoechst 33342, 100 nM MitoTracker Deep Red, and either DMSO or 10 μM PalmB, washed, loaded with 250 nM mitoDPP-2 for 10 min, and then analyzed by confocal fluorescence microscopy. Images for MitoTracker, mitoDPP-2, Hoechst 33342 nuclear stain, brightfield, and an overlay of MitoTracker, mitoDPP-2, and Hoechst 33342 are shown for each set of conditions. Three representative images are shown for each condition. 5 μm scale bar shown.

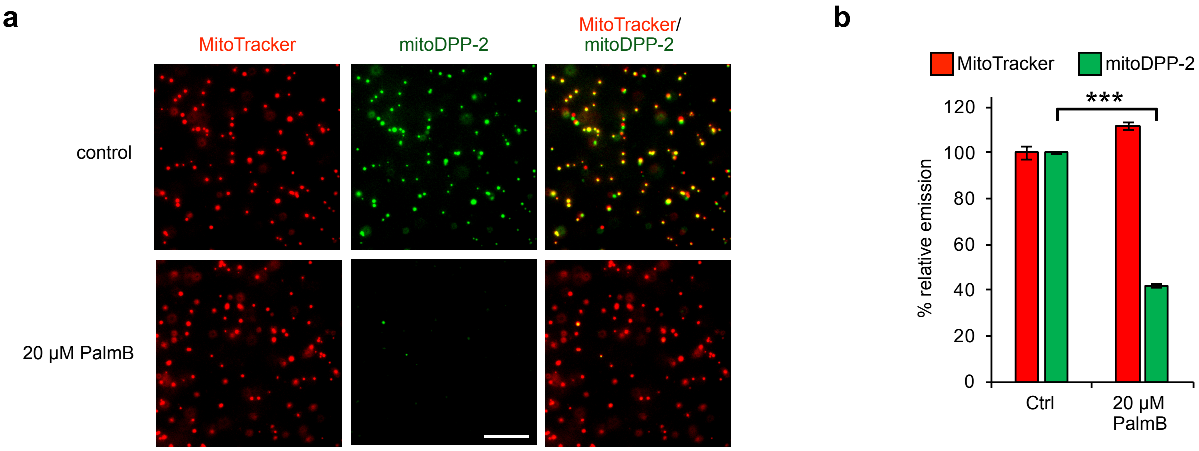

Supplementary Figure 6: Epifluorescence imaging of live mitochondria with mitoDPP-2. (**a**) Live mitochondria pretreated with PalmB and MitoTracker were imaged after 10 min incubation with 1 μM mitoDPP-2. Images for MitoTracker, mitoDPP-2 and an overlay of MitoTracker and mitoDPP-2 are shown for each set of conditions. 20 μm scale bar shown. (**b**) Quantification of images shown in (**a**). For plots, statistical analyses performed with a two-tailed Student's *t*-test with unequal variance, *** P value < 10^{-5}; n = 3 (for **b**), error bars are ± s.e.m.

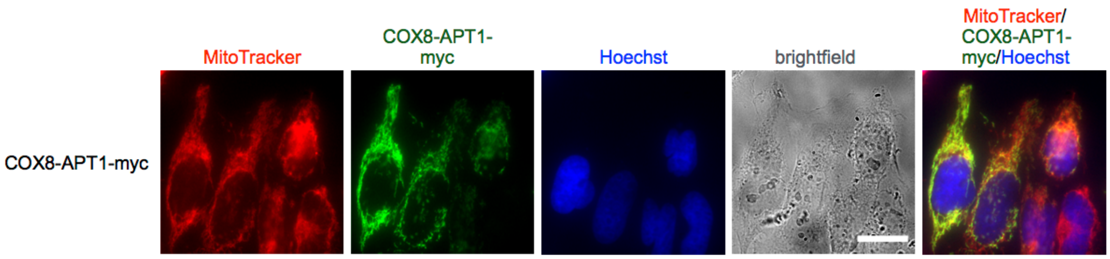

Supplementary Figure 7: Complete imaging series from Fig. 4a. Immunostaining of HeLa cells transfected with COX8-APT1-myc for 26 h analyzed by epifluorescence microscopy. Images for MitoTracker, COX8-APT1-myc (GFP), Hoechst 33342 nuclear stain, brightfield, and an overlay of MitoTracker, GFP, and Hoechst 33342 are shown for each set of conditions. 20 μm scale bar shown.

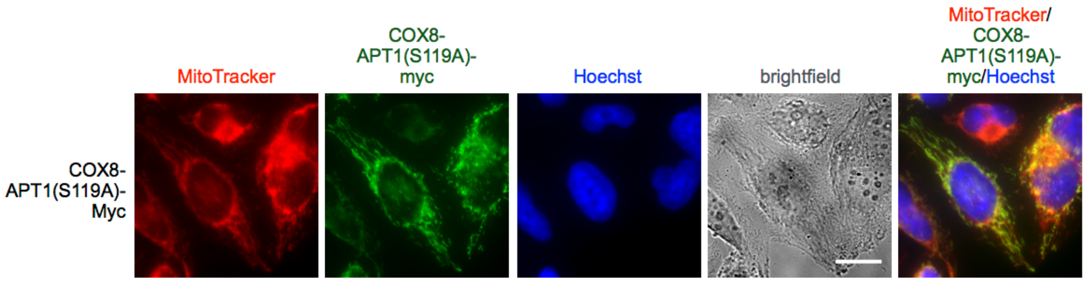

Supplementary Figure 8: Localization of inactive mutant of COX8-APT1-myc. Immunostaining of HeLa cells transfected with COX8-APT1(S119A)-myc for 26 h analyzed by epifluorescence microscopy. Images for MitoTracker, COX8-APT1(S119A)-myc (GFP), Hoechst 33342 nuclear stain, brightfield, and an overlay of MitoTracker, GFP, and Hoechst 33342 are shown for each set of conditions. 20 μm scale bar shown.

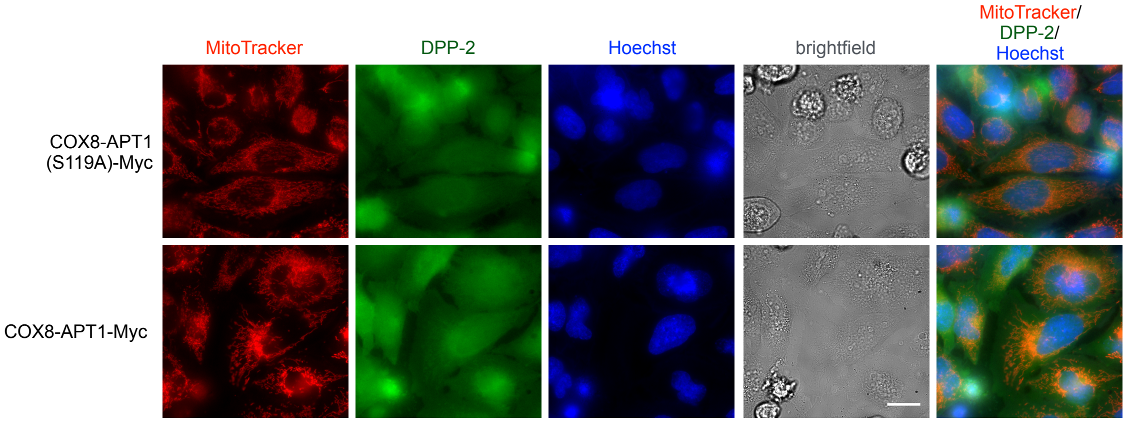

Supplementary Figure 9: Complete imaging series from Fig. 4c. HeLa cells transfected with COX8-APT1-myc or inactive mutant for 30 h were treated for 30 min with 1 μM Hoechst 33342, 100 nM MitoTracker Deep Red, and washed, loaded with 1μM DPP-2 for 10 min, and then analyzed by epifluorescence microscopy. Images for MitoTracker, DPP-2, Hoechst 33342 nuclear stain, brightfield, and an overlay of MitoTracker, DPP-2, and Hoechst 33342 are shown for each set of conditions. 20 μm scale bar shown.

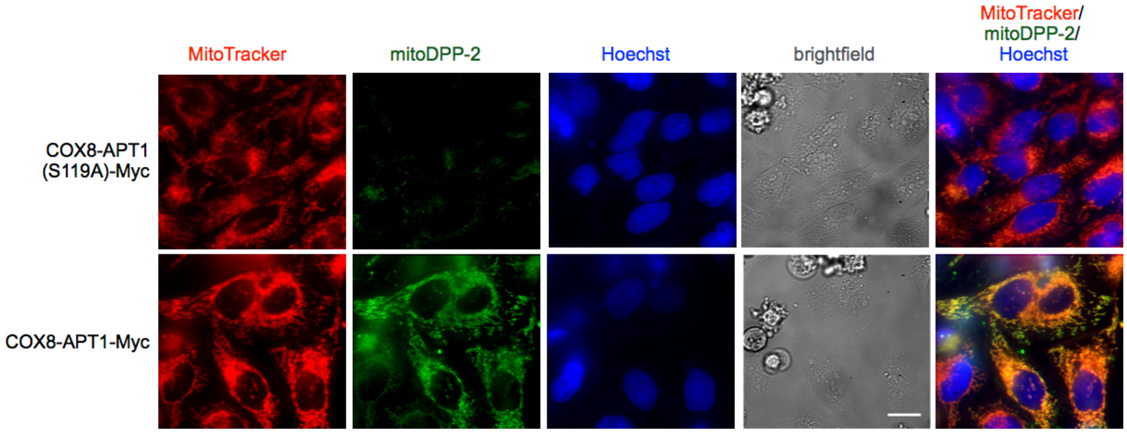

Supplementary Figure 10: Complete imaging series from Fig. 4e. HeLa cells transfected with COX8-APT1-myc or inactive mutant for 30 h were treated for 30 min with 1 μM Hoechst 33342, 100 nM MitoTracker Deep Red, and washed, loaded with 500 nM mitoDPP-2 for 10 min, and then analyzed by epifluorescence microscopy. Images for MitoTracker, mitoDPP-2, Hoechst 33342 nuclear stain, brightfield, and an overlay of MitoTracker, mitoDPP-2, and Hoechst 33342 are shown for each set of conditions. 20 μm scale bar shown.

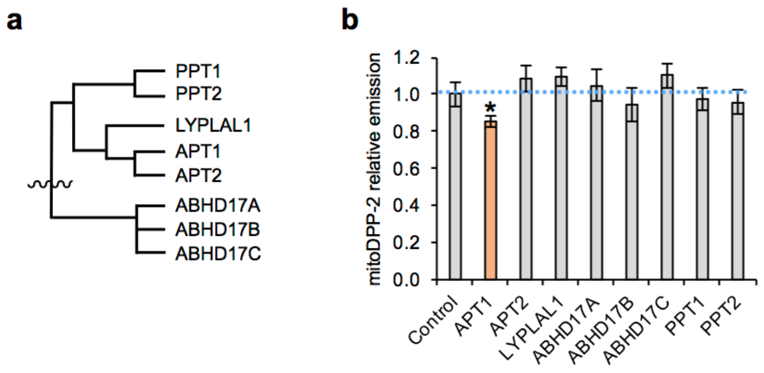

Supplementary Figure 11: Discovering putative mitochondrial *S*-deacylases (a) Relationship of genes targeted in RNAi screen as potential mitochondrial *S*-deacylases. (b) HEK293T cells transfected with pooled shRNA libraries targeting each identified gene by RNAi for 52-56 h. The cells were then washed, loaded with 500 nM mitoDPP-2, and then analyzed for fluorescence output by plate reader. For plots, statistical analyses performed with a two-tailed Student's t-test with unequal variance, * P value < 0.05; n = 3, error bars are ± s.e.m.

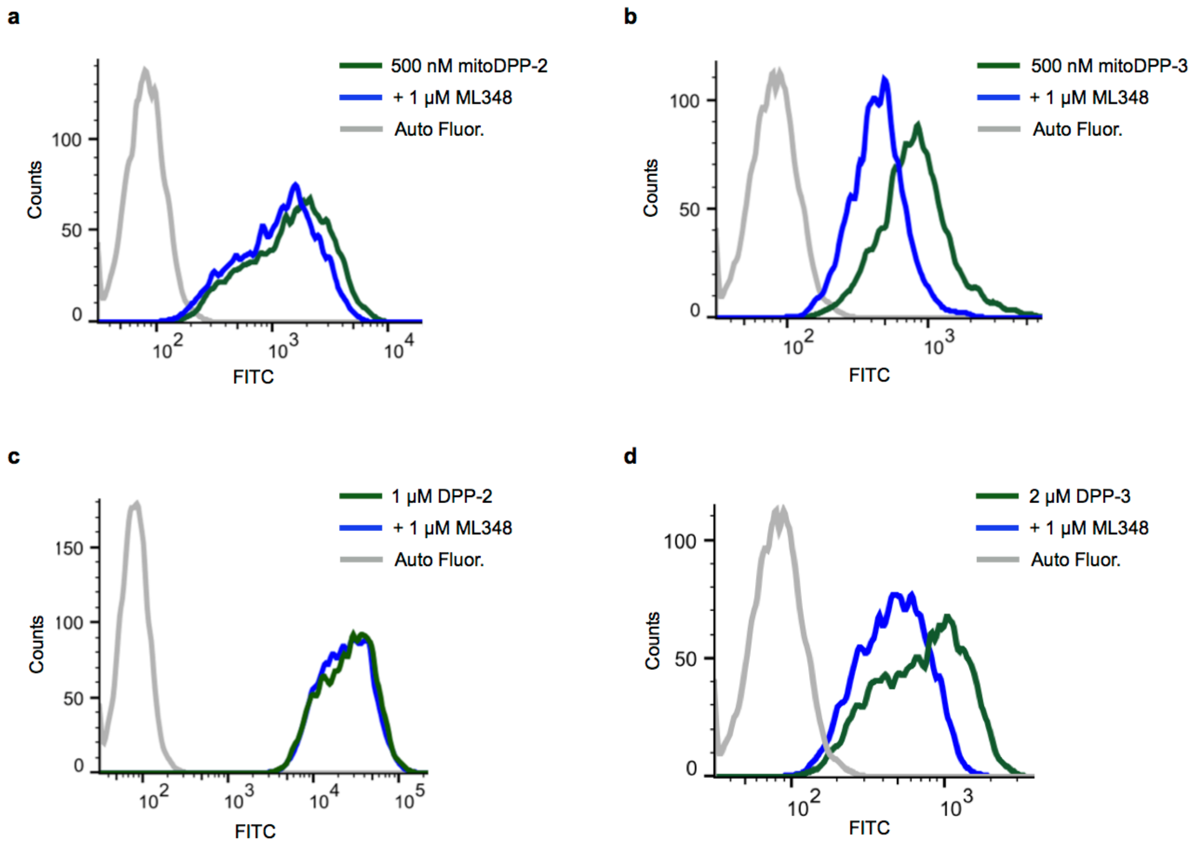

Supplementary Figure 12: Response of mitoDPPs and DPPs to ML348 inhibition analyzed by flow cytometry. HEK293T cells pretreated for 30 min with DMSO or 1 μM ML348 treated with (**a**) 500 nM mitoDPP-2, (**b**) 500 nM mitoDPP-3, (**c**) 1 μM DPP-2, or (**d**) 2 μM DPP-3. 1 μM ML348 affects signal of mitoDPP-2 slightly in comparison to its cysolic analog DPP-2. However, ML348 significantly reduces signal from both mitoDPP-3 and DPP-3 potentially reporting mitochondrial and cytosolic pools of APT1.

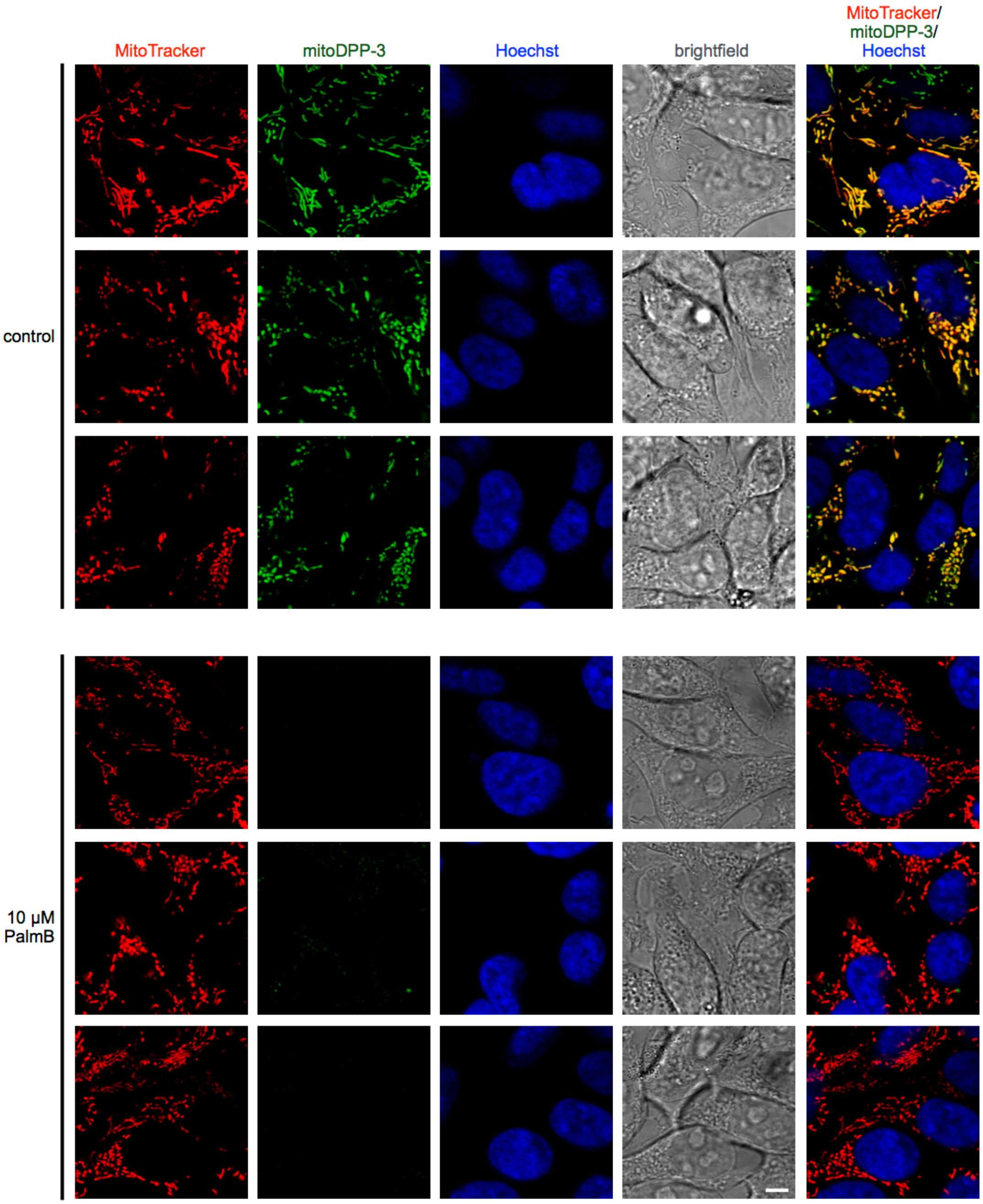

Supplementary Figure 13: Complete imaging series from Fig. 6a. HEK293T cells treated for 30 min with 1 μM Hoechst 33342, 100 nM MitoTracker Deep Red, and either DMSO or 10 μM PalmB, washed, loaded with 324 nM mitoDPP-3 for 10 min, and then analyzed by confocal fluorescence microscopy. Images for MitoTracker, mitoDPP-3, Hoechst 33342 nuclear stain, brightfield, and an overlay of Mitotracker, mitoDPP-3, and Hoechst 33342 are shown for each set of conditions. Three representative images are shown for each condition. 5 μm scale bar shown.

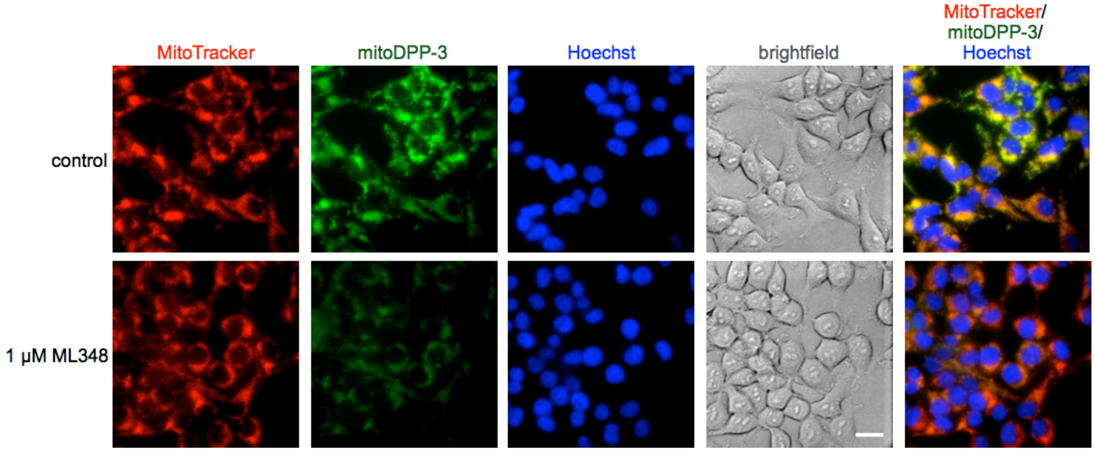

Supplementary Figure 14: Complete imaging series from Fig. 6d. HEK293T cells treated for 30 min with 1 μM Hoechst 33342, 100 nM MitoTracker Deep Red, and either DMSO or 1 μM ML348, washed, loaded with 324 nM mitoDPP-3 with DMSO or 1 μM ML348 for 10 min, and then analyzed by epifluorescence microscopy. Images for MitoTracker, mitoDPP-3, Hoechst 33342 nuclear stain, brightfield, and an overlay of Mitotracker, mitoDPP-3, and Hoechst 33342 are shown for each set of conditions. 20 μm scale bar shown.

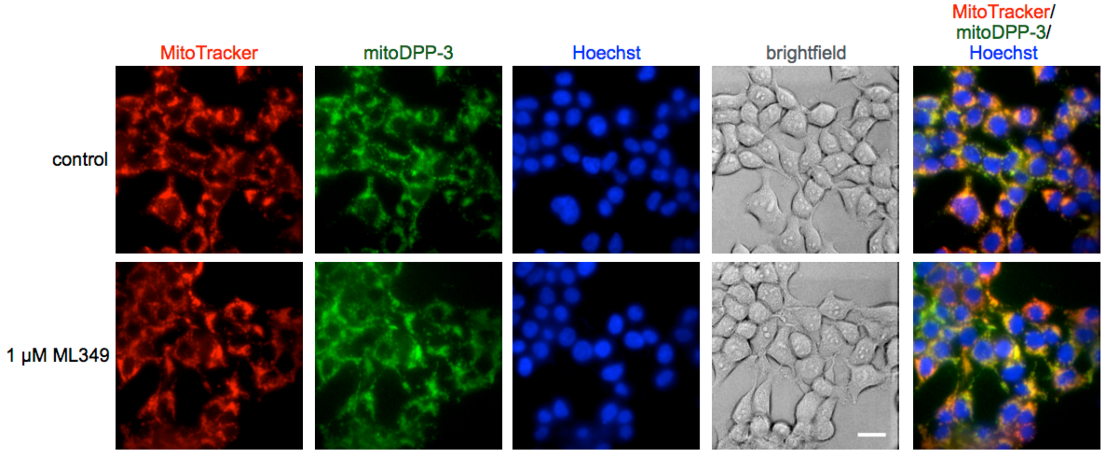

Supplementary Figure 15: Complete imaging series from Fig. 6e. HEK293T cells treated for 30 min with 1 μM Hoechst 33342, 100 nM MitoTracker Deep Red, and either DMSO or 1 μM ML349, washed, loaded with 324 nM mitoDPP-3 with DMSO or 1μM ML349 for 10 min, and then analyzed by epifluorescence microscopy. Images for MitoTracker, mitoDPP-3, Hoechst 33342 nuclear stain, brightfield, and an overlay of Mitotracker, mitoDPP-3, and Hoechst 33342 are shown for each set of conditions. 20 μm scale bar shown.

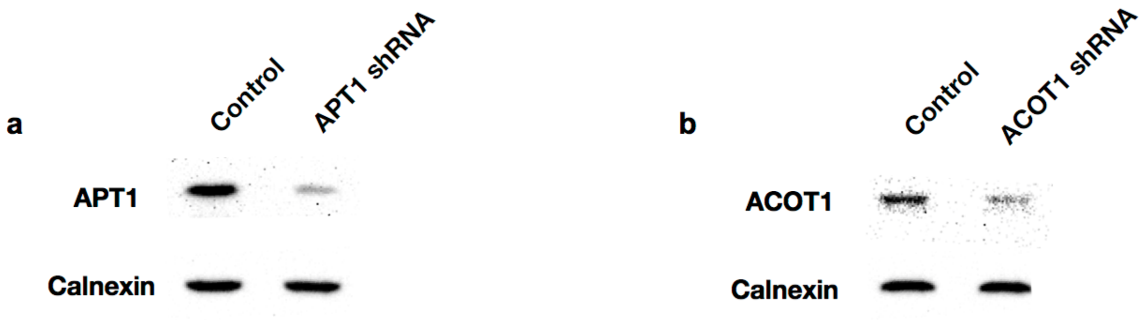

Supplementary Figure 16: Knockdown efficiency analyzed by western blot. (**a**) APT1 and (**b**) ACOT1 vectors or control vectors transfected into HEK293T cells and then analyzed by western blot. Calnexin was used as an internal control.

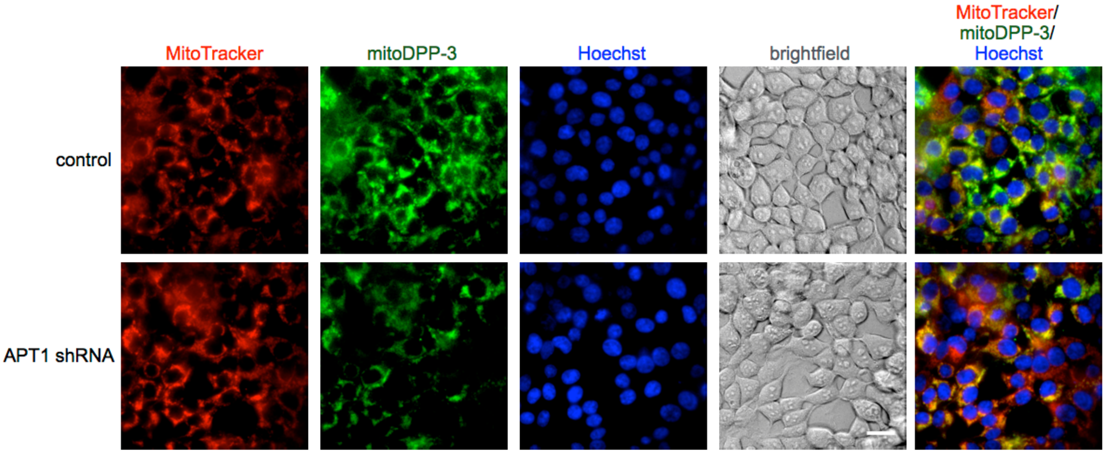

Supplementary Figure 17: Complete imaging series from Fig. 6f. After treatment with either shRNA(control) or shRNA(APT1) for 35 hours, HEK293T cells treated for 30 min with 1 μM Hoechst 33342, 100 nM MitoTracker Deep Red, washed, loaded with 324 nM mitoDPP-3 for 10 min, and then analyzed by epifluorescence microscopy. Images for MitoTracker, mitoDPP-3, Hoechst 33342 nuclear stain, brightfield, and an overlay of Mitotracker, mitoDPP-3, and Hoechst 33342 are shown for each set of conditions. 20 μm scale bar shown.

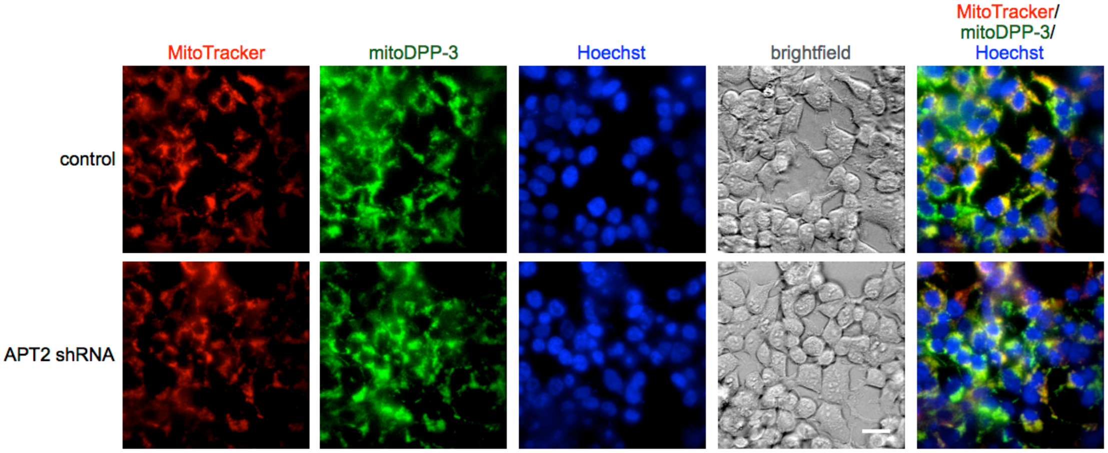

Supplementary Figure 18: Complete imaging series from Fig. 6g. After treatment with either shRNA(control) or shRNA(APT2) for 35 hours, HEK293T cells treated for 30 min with 1 μM Hoechst 33342, 100 nM MitoTracker Deep Red, washed, loaded with 324 nM mitoDPP-3 for 10 min, and then analyzed by epifluorescence microscopy. Images for MitoTracker, mitoDPP-3, Hoechst 33342 nuclear stain, brightfield, and an overlay of Mitotracker, mitoDPP-3, and Hoechst 33342 are shown for each set of conditions. 20 μm scale bar shown.

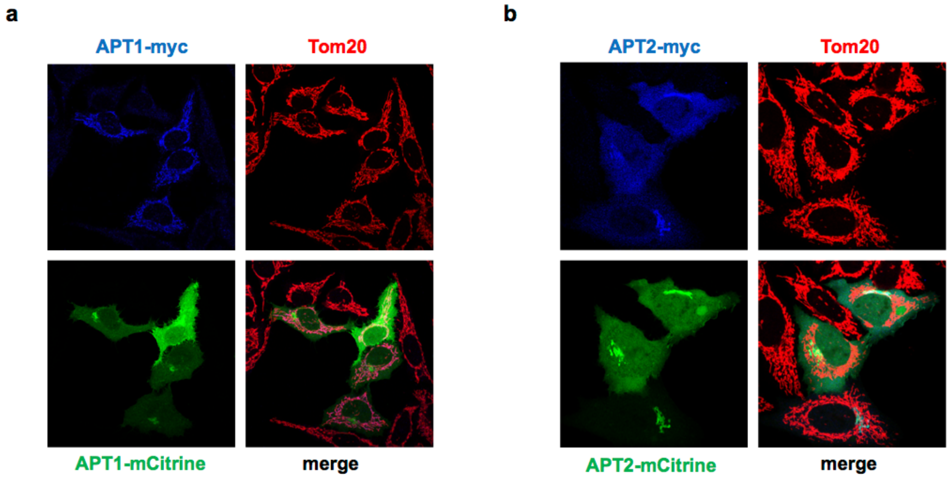

Supplementary Figure 19: Co-expression of APT1 or APT2 fused to a C-terminal myc tag and a mCitrine tag. (a) Immunostaining of HeLa cells after 48 hours of transfection with C-terminal myc (blue) and m-Citrine (green) tagged to human APT1. Mitochondrial protein Tom20 (red) shows APT1-myc is primarily localized in mitochondria and that the mCitrine tag affects APT1 localization. (**b**) Immunostaining of HeLa cells after 48 hours of transfection with C-terminal myc (blue) and m-Citrine (green) tagged to human APT2. mCitrine does not affect localization of APT2.

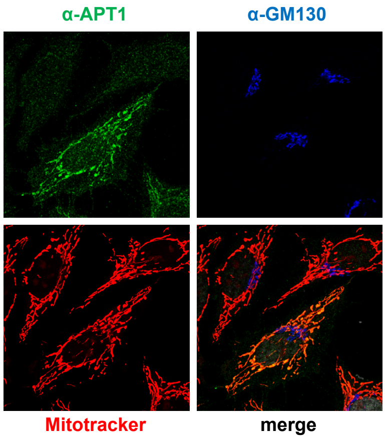

Supplementary Figure 20: Mitochondrial localization of untagged human APT1. Immunostaining of HeLa cells after 48 hours of transfection with a vector containing a stop codon between APT1 and mCitrine (APT1-Stop-mCitrine) demonstrates predominant mitochondrial localization when compared with MitoTracker. Images for APT1 (Green), MitoTracker (Red), Golgi Marker (GM130; Blue) and merged channels are shown.

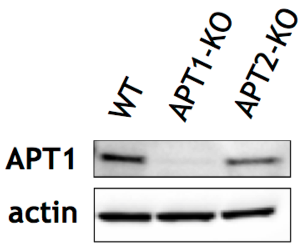

Supplementary Figure 21: Efficiency of APT1 KO in HAP1 cells analyzed by western blot. Cell lysates from WT, APT1-KO and APT2-KO HAP1 cell were analyzed by western blot using an anti-APT1/LYPLA1 antibody. Actin was used a loading control.

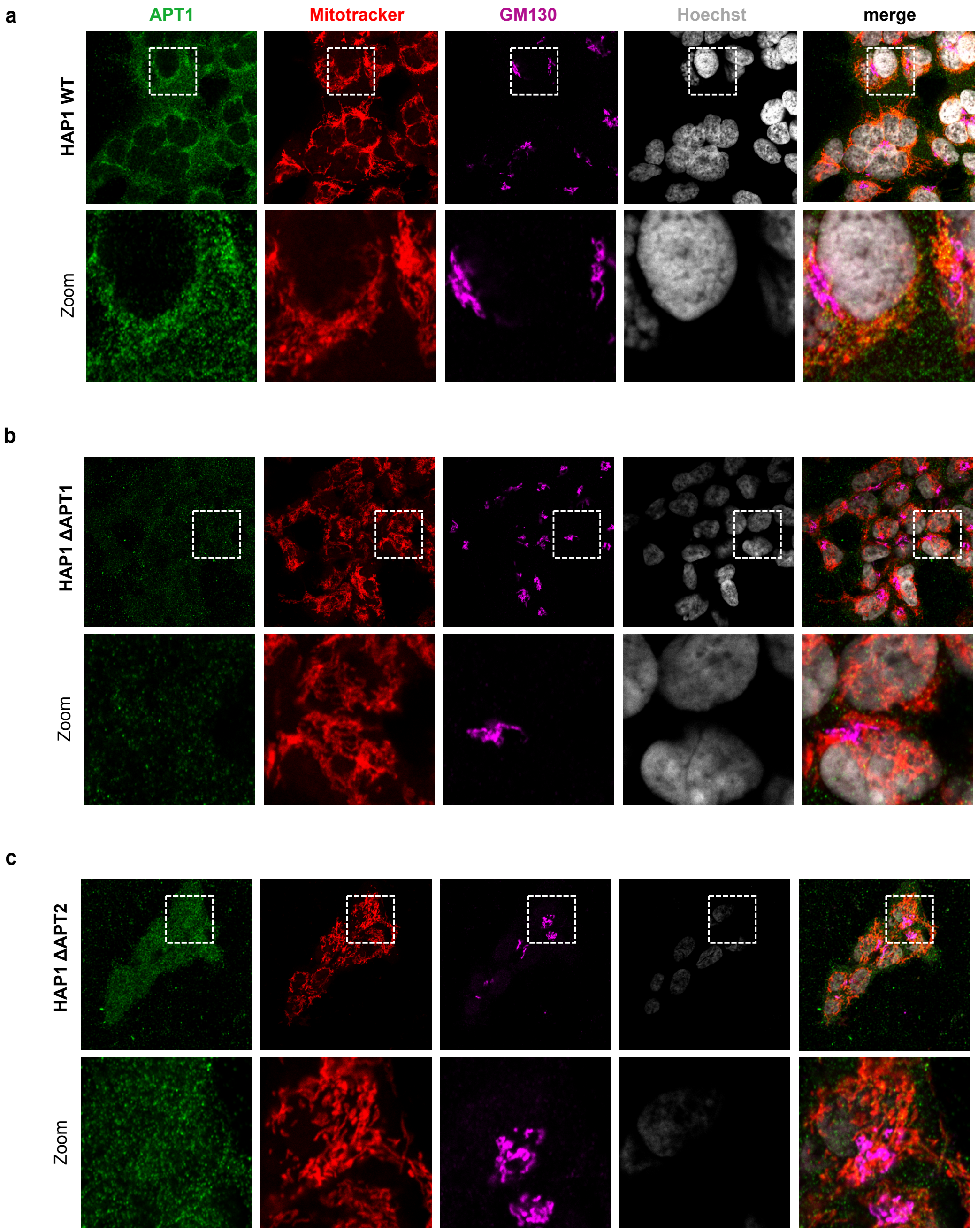

Supplementary Figure 22: Immunostaining of endogeneous APT1 in WT, APT1-KO and APT2-KO HAP1 cells. (**a**) Immunostaining for APT1 (Green) in WT HAP1 cells shows signal which colocalizes with MitoTracker (Red). (**b**) The signal in the green channel that colocalizes with MitoTracker is lost in APT1-KO HAP1 cells. (**c**) APT2-KO HAP1 cells, however, maintain the signal in green channel as in the case of WT HAP1 cells. Images for APT1 (Green), MitoTracker (Red), Golgi Marker (GM130; Pink), Nuclear marker (Hoechst 33342; Grey) and merged channels are shown for each set of conditions.

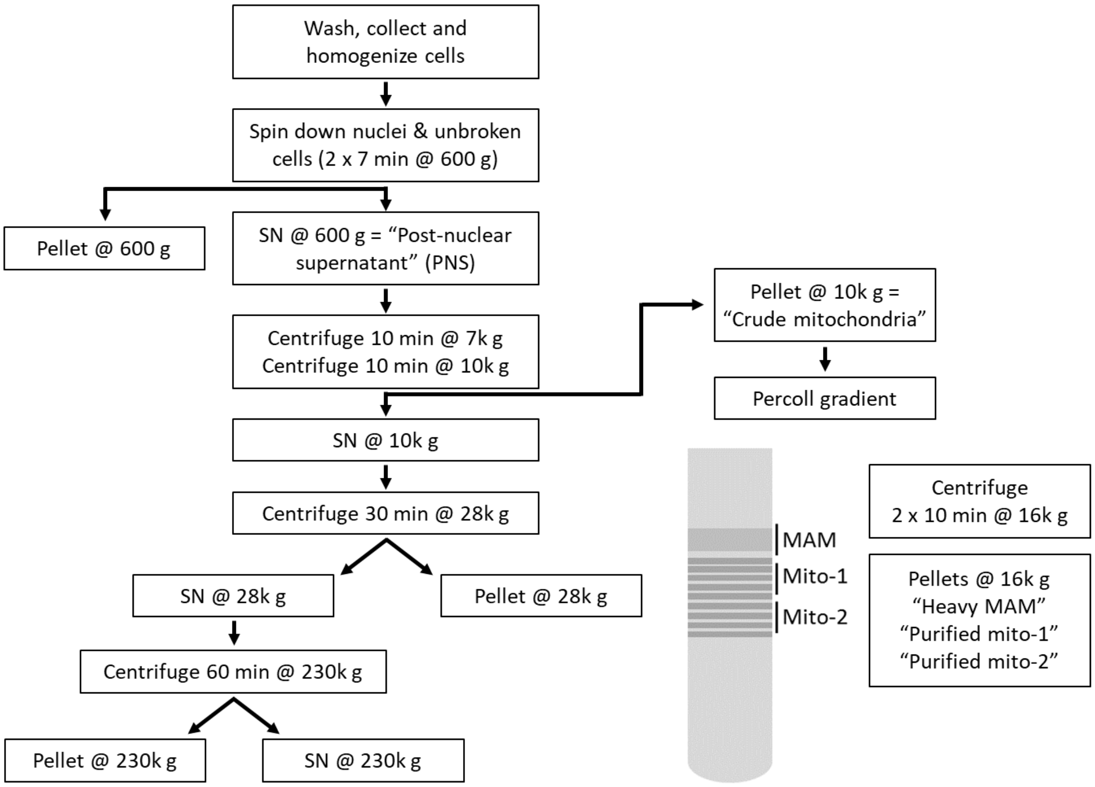

Supplementary Figure 23: Scheme of the subcellular fractionation protocol used to recover purified mitochondria fractions from HeLa cells (adapted from Wieckowski et al[1]). SN: supernatant; MAM: mitochondria-associated ER membranes.

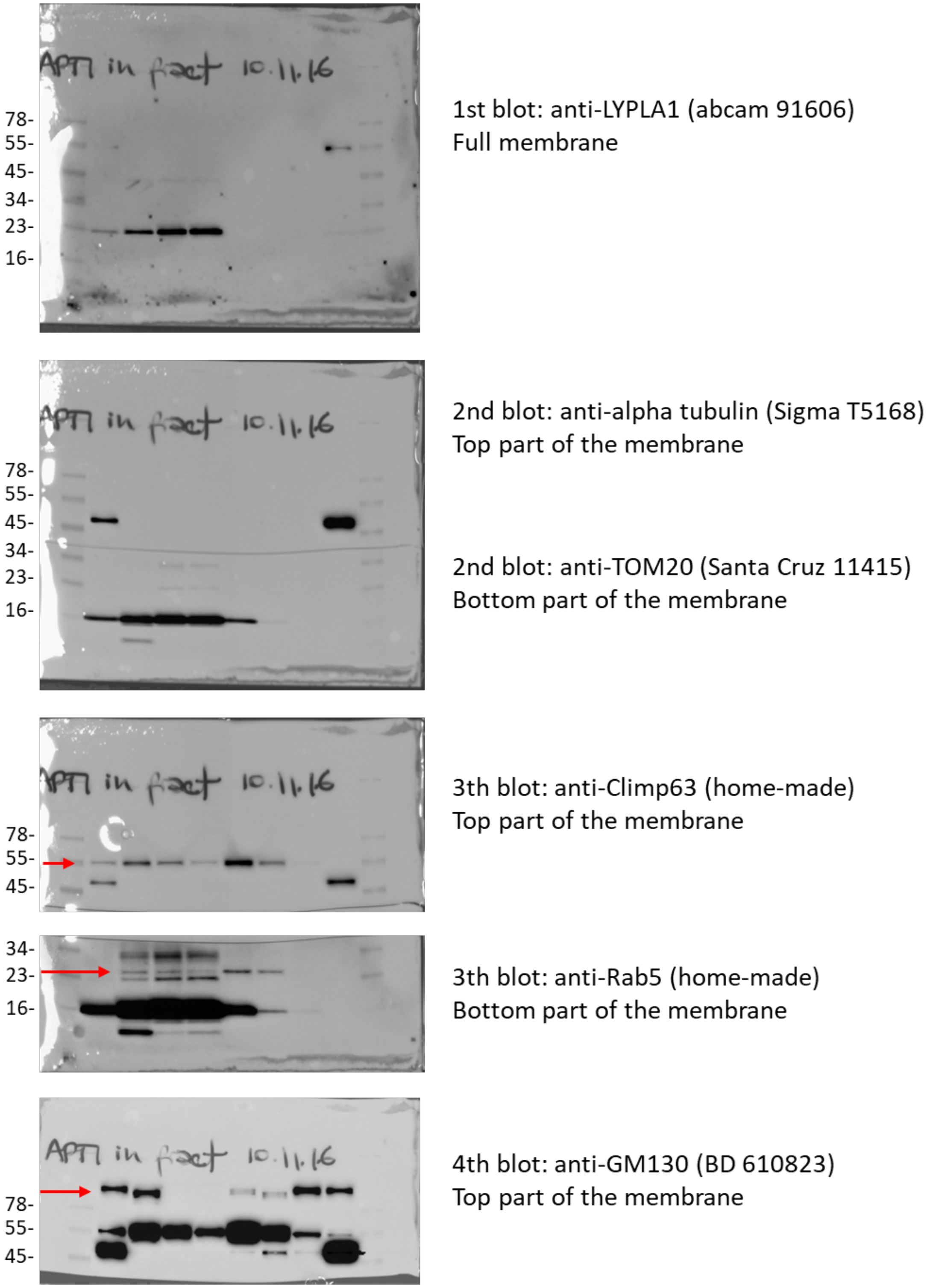

Supplementary Figure 24: Original uncropped western blots corresponding to Figure 7c. Red arrows point at the right molecular weight to be considered in each case. Additional bands correspond to previous blottings on the same membrane. Numbers on the left indicate protein sizes of the corresponding bands of the molecular weight marker (first lane). LYPLA1 = APT1.

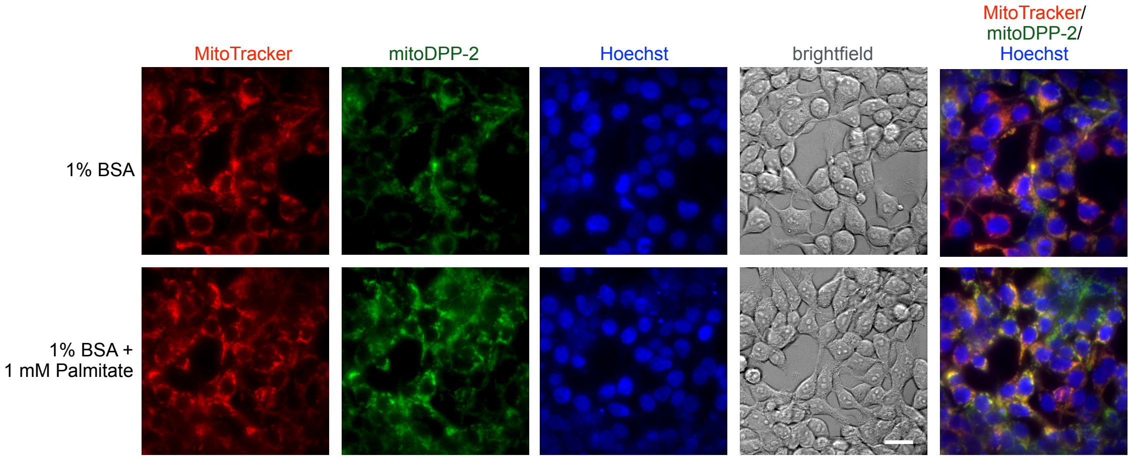

Supplementary Figure 25: Complete imaging series from Fig. 8. After treatment for 6 hours with either 1% BSA or 1% BSA with 1 mM Palmitate post 6 hours of starvation, HEK293T cells treated for 30 min with 1 μM Hoechst 33342, 100 nM MitoTracker Deep Red, washed, loaded with 500 nM mitoDPP-2 for 10 min, and then analyzed by epifluorescence microscopy. Images for MitoTracker, mitoDPP-2, Hoechst 33342 nuclear stain, brightfield, and an overlay of Mitotracker, mitoDPP-2, and Hoechst 33342 are shown for each set of conditions. 20 μm scale bar shown.

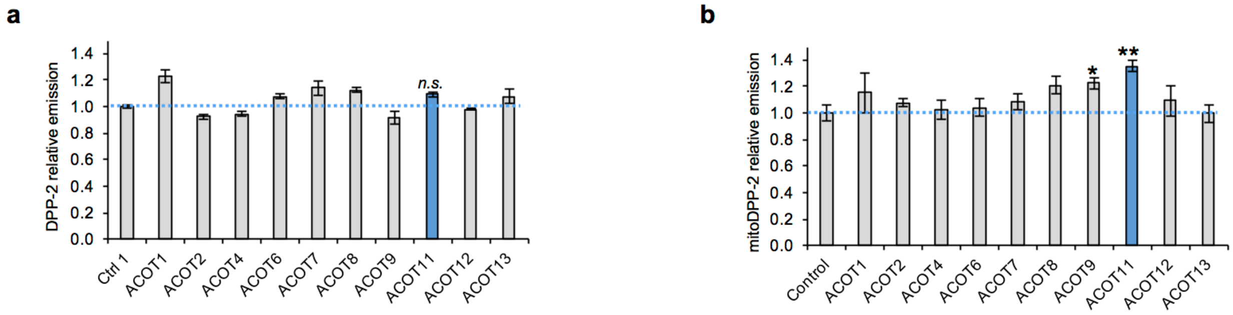

Supplementary Figure 26: Potential interplay between lipid homeostasis and cytosolic/mitochondrial *S*-deacylase activities investigated by perturbation of ACOTs. HEK293T cells transfected with pooled shRNA libraries targeting each identified ACOT by RNAi for 58 h. The cells were then washed, loaded with 1 μM DPP-2 (**a**) or 500 nM mitoDPP-2 (**b**), and then analyzed by fluorescence plate reader. For all plots, statistical analyses performed with a two-tailed Student's *t*-test with unequal variance, * P value < 0.05; ** P value < 0.007, n = 3 and error bars are ± s.e.m.

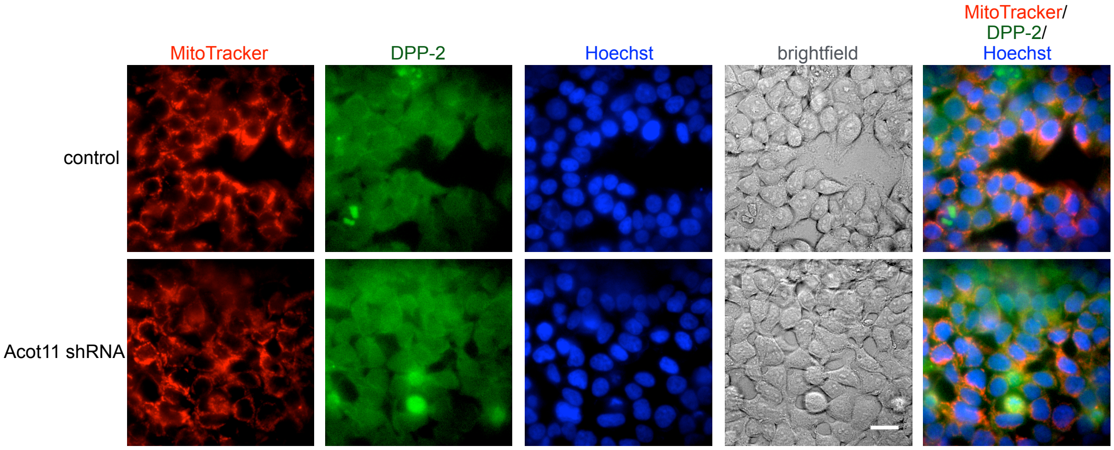

Supplementary Figure 27: Complete imaging series from Fig. 9a. After treatment with either shRNA(control) or shRNA(ACOT11) for 56 hours, HEK293T cells treated for 30 min with 1 μM Hoechst 33342, 100 nM MitoTracker Deep Red, washed, loaded with 1 μM DPP-2 for 10 min, and then analyzed by epifluorescence microscopy. Images for MitoTracker, DPP-2, Hoechst 33342 nuclear stain, brightfield, and an overlay of Mitotracker, DPP-2, and Hoechst 33342 are shown for each set of conditions. 20 μm scale bar shown.

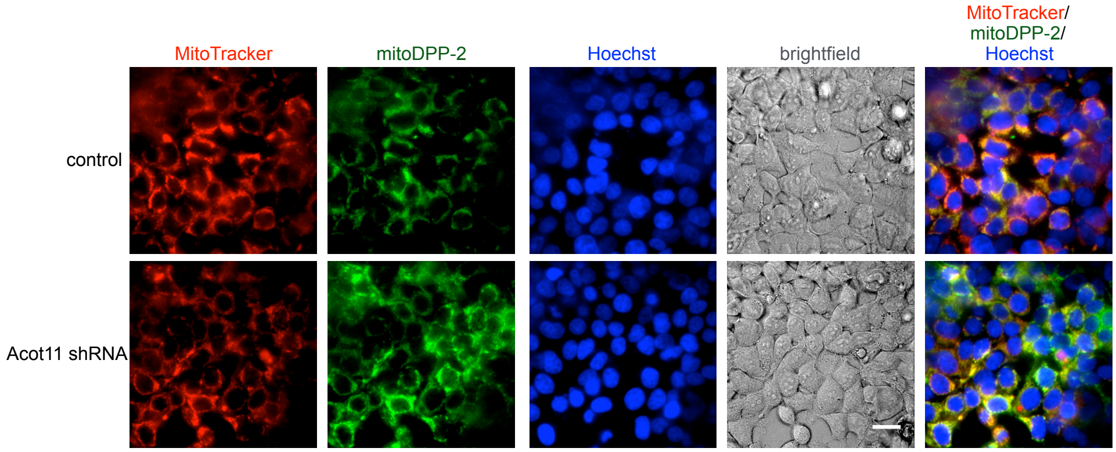

Supplementary Figure 28: Complete imaging series from Fig. 9c. After treatment with either shRNA(control) or shRNA(ACOT11) for 56 hours, HEK293T cells treated for 30 min with 1 μM Hoechst 33342, 100 nM MitoTracker Deep Red, washed, loaded with 500 nM mitoDPP-2 for 10 min, and then analyzed by epifluorescence microscopy. Images for MitoTracker, mitoDPP-2, Hoechst 33342 nuclear stain, brightfield, and an overlay of Mitotracker, mitoDPP-2, and Hoechst 33342 are shown for each set of conditions. 20 μm scale bar shown.

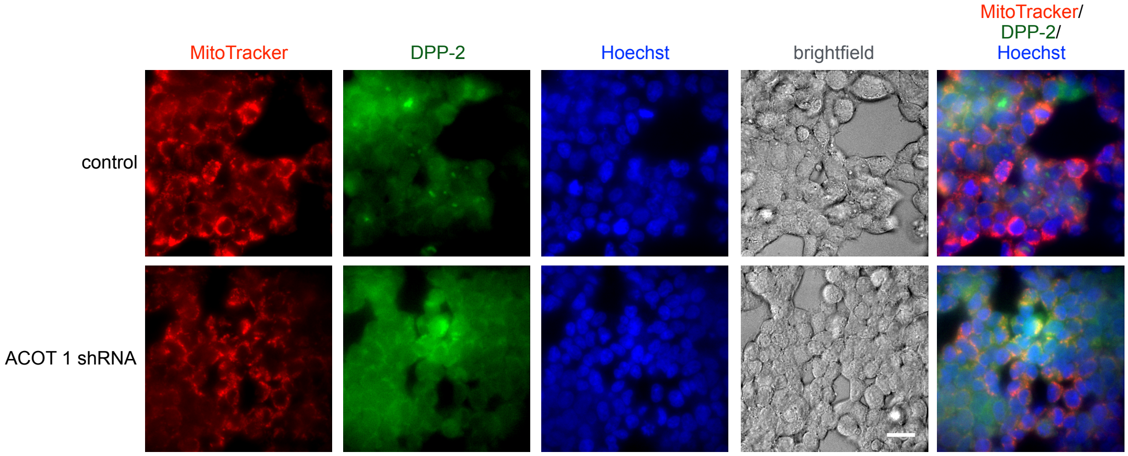

Supplementary Figure 29: Complete imaging series from Fig. 9e. After treatment with either shRNA(control) or shRNA(ACOT1) for 58 hours, HEK293T cells treated for 30 min with 1 μM Hoechst 33342, 100 nM MitoTracker Deep Red, washed, loaded with 1 μM DPP-2 for 10 min, and then analyzed by epifluorescence microscopy. Images for MitoTracker, DPP-2, Hoechst 33342 nuclear stain, brightfield, and an overlay of Mitotracker, DPP-2, and Hoechst 33342 are shown for each set of conditions. 20 μm scale bar shown.

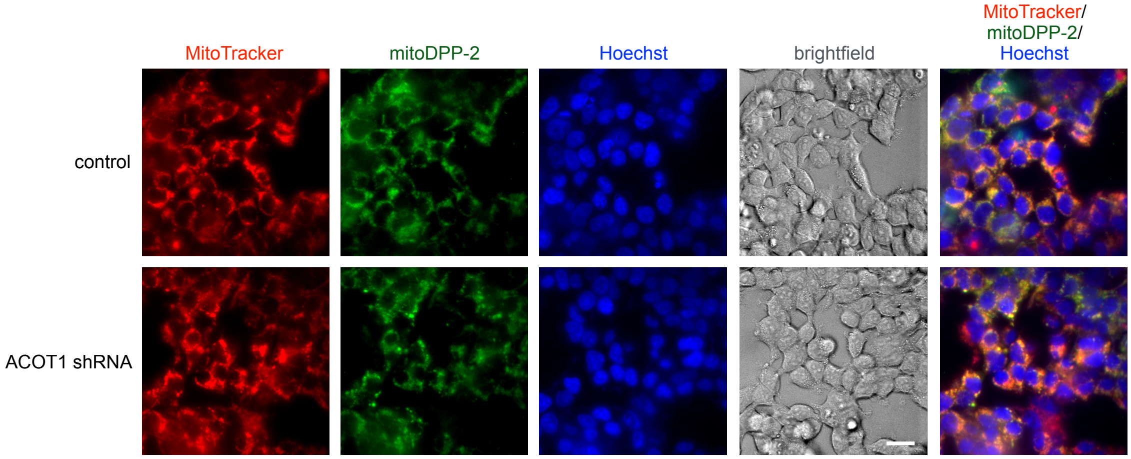

Supplementary Figure 30: Complete imaging series from Fig. 9g. After treatment with either shRNA(control) or shRNA(ACOT1) for 58 hours, HEK293T cells treated for 30 min with 1 μM Hoechst 33342, 100 nM MitoTracker Deep Red, washed, loaded with 500 nM mitoDPP-2 for 10 min, and then analyzed by epifluorescence microscopy. Images for MitoTracker, mitoDPP-2, Hoechst 33342 nuclear stain, brightfield, and an overlay of Mitotracker, mitoDPP-2, and Hoechst 33342 are shown for each set of conditions. 20 μm scale bar shown.

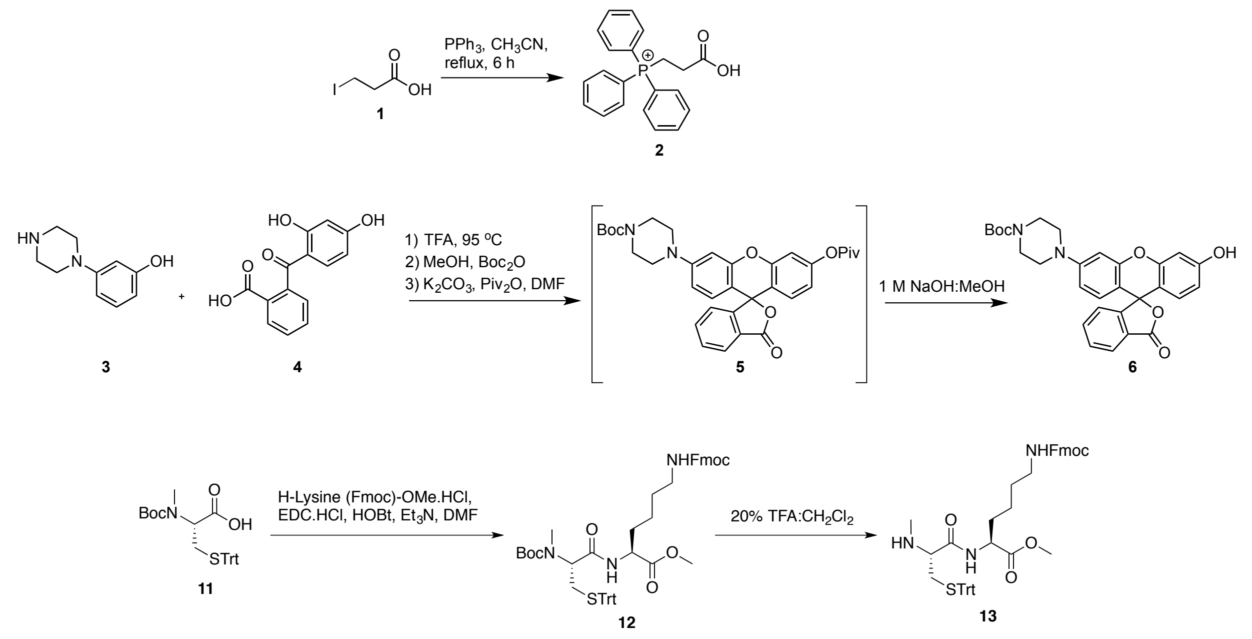

Supplementary Figure 31: Synthetic schemes for **2**, **6**, and **13.**

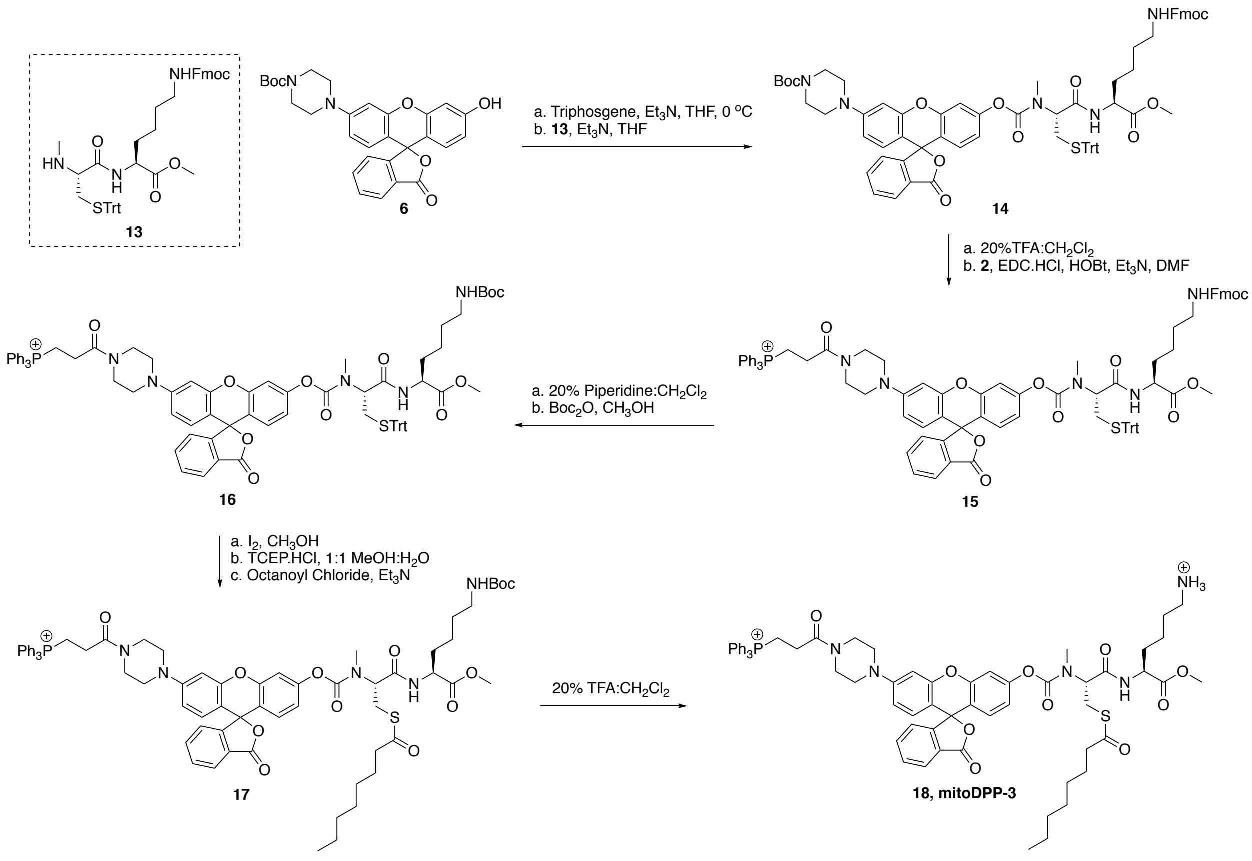

Supplementary Figure 32: Synthetic scheme for **18** (**mitoDPP-3**).

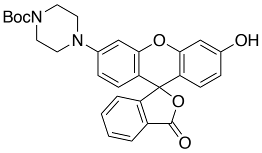

^{1}H-NMR (500 MHz; $CDCl_3$)

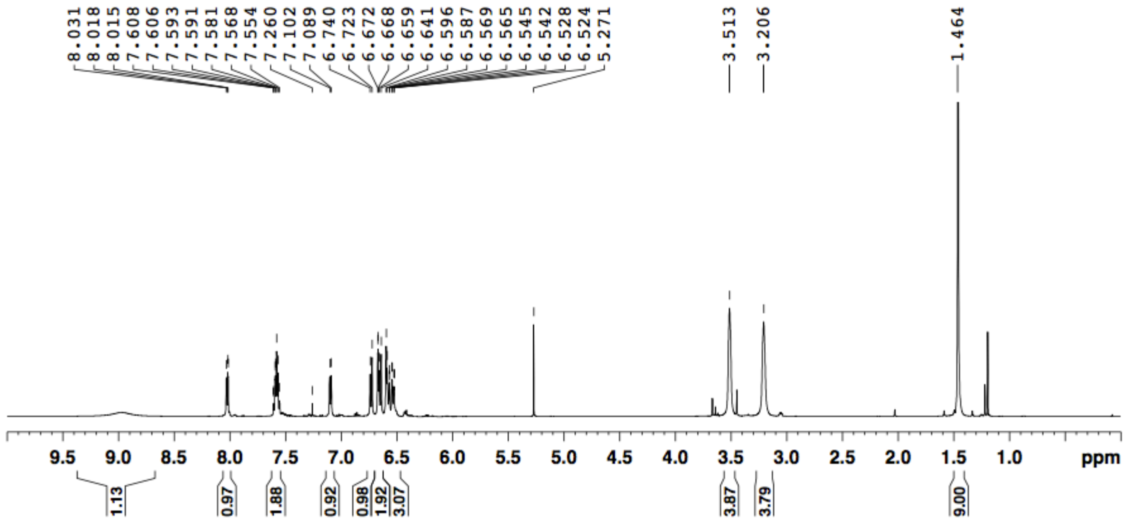

^{13}C-NMR (126 MHz; $CDCl_3$)

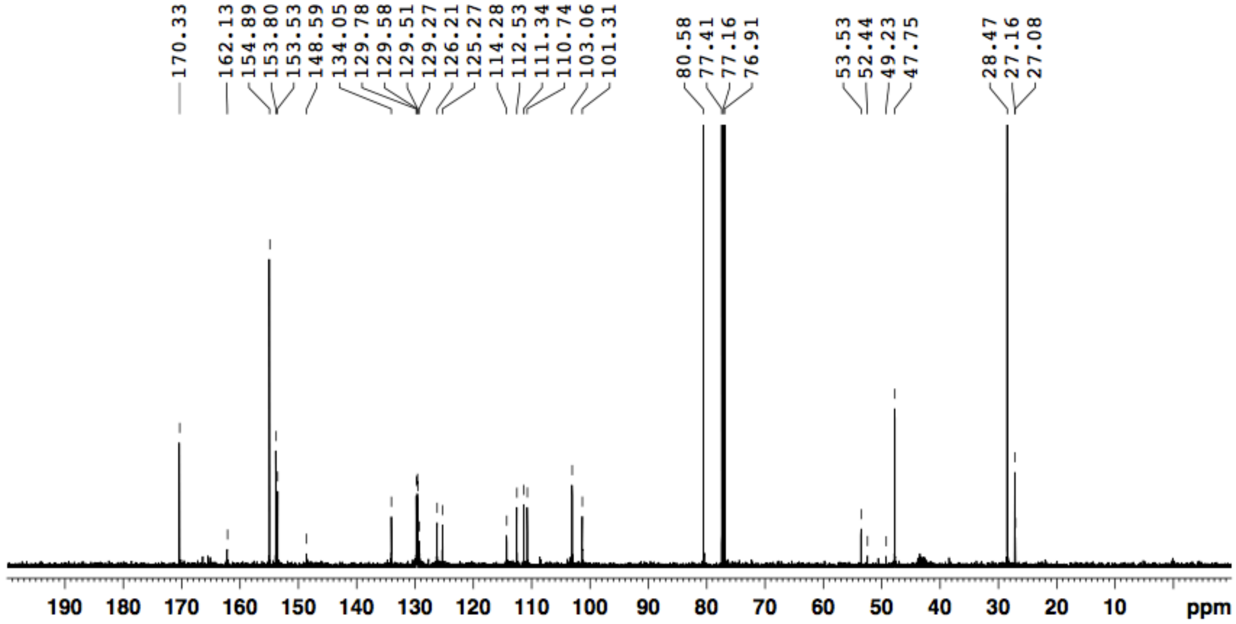

Supplementary Figure 33: ^{1}H-NMR and ^{13}C-NMR of 6.

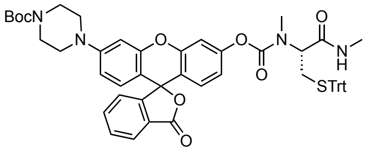

^{1}H-NMR (500 MHz; $CDCl_3$)

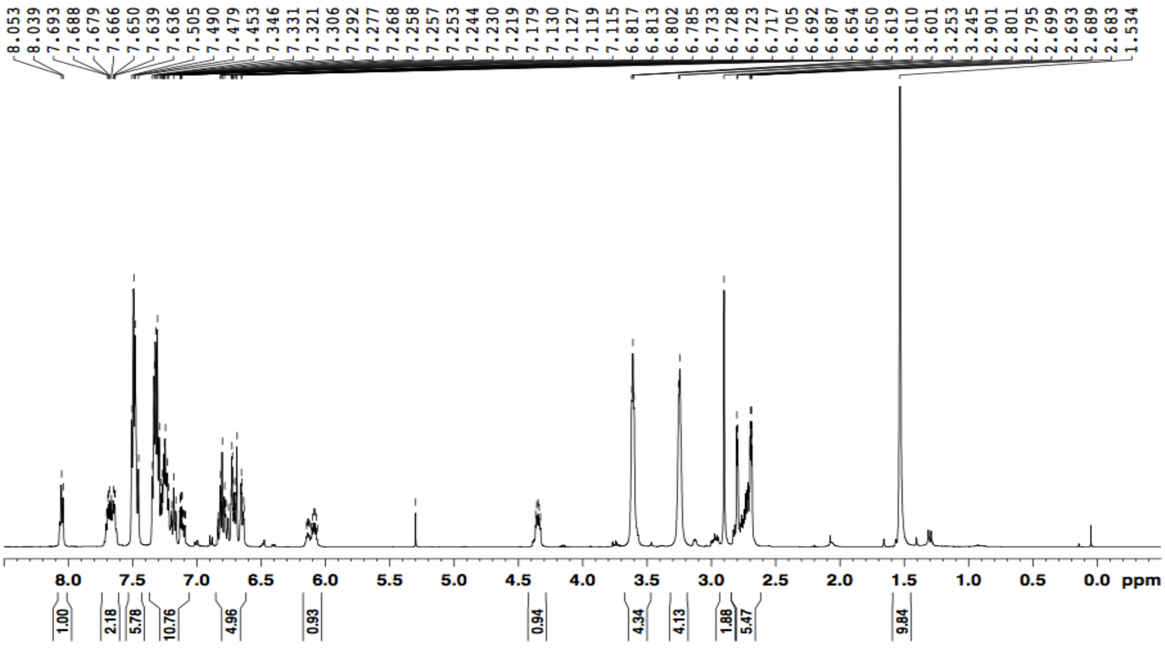

^{13}C-NMR (126 MHz; $CDCl_3$)

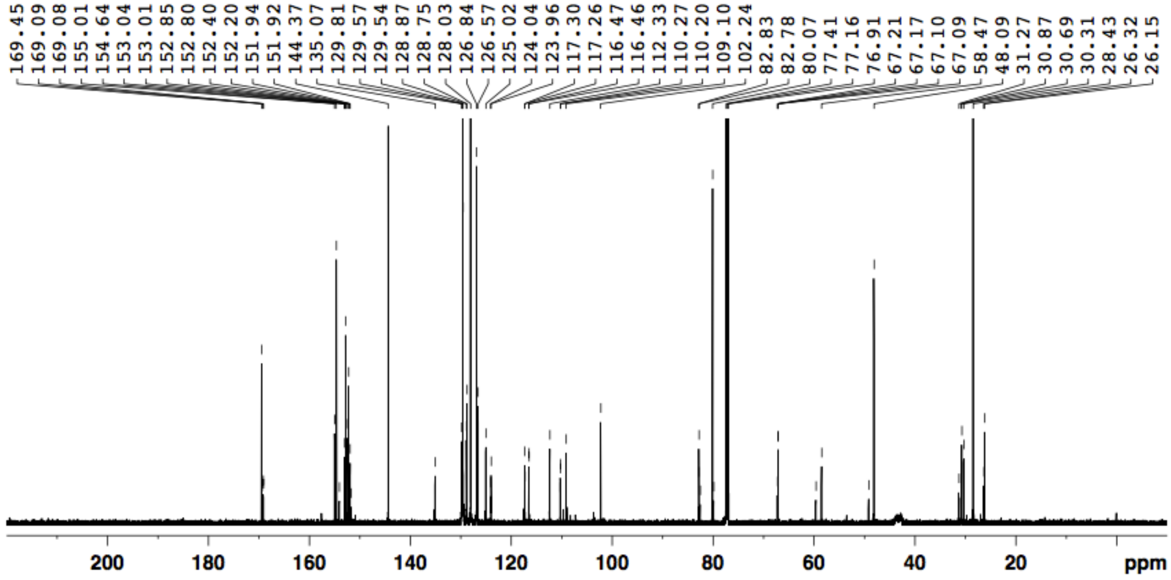

Supplementary Figure 34: ^{1}H-NMR and ^{13}C-NMR of 8.

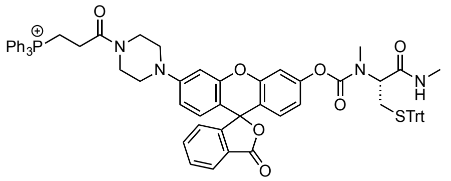

^{1}H-NMR (500 MHz; $CDCl_3$)

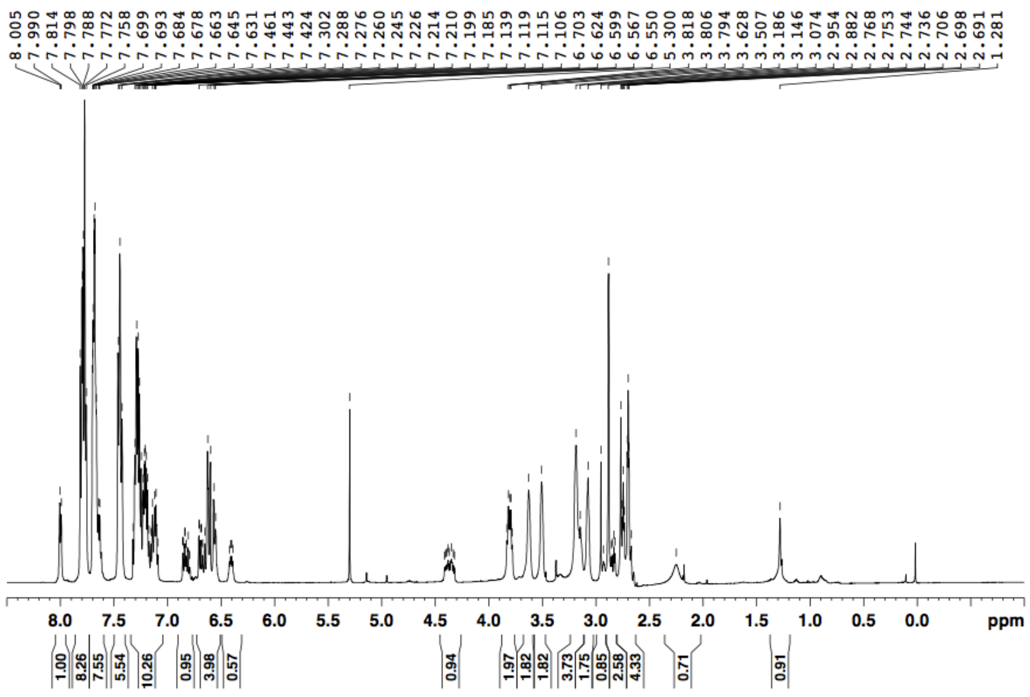

^{13}C-NMR (126 MHz; $CDCl_3$)

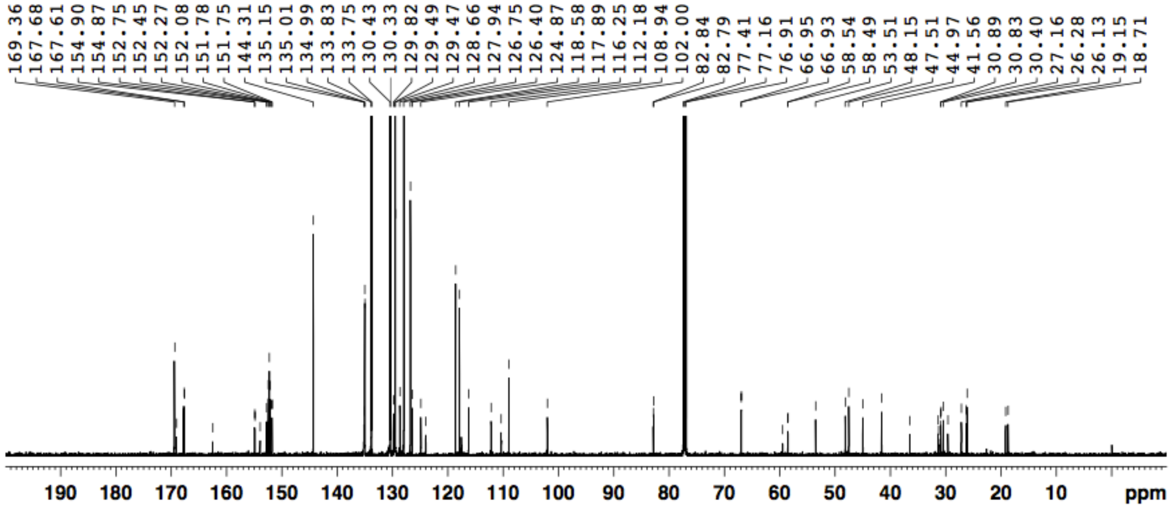

Supplementary Figure 35: ^{1}H-NMR and ^{13}C-NMR of 9.

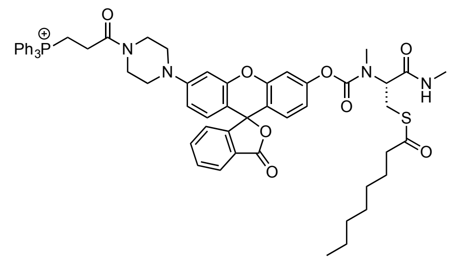

^{1}H-NMR (500 MHz; $CDCl_3$)

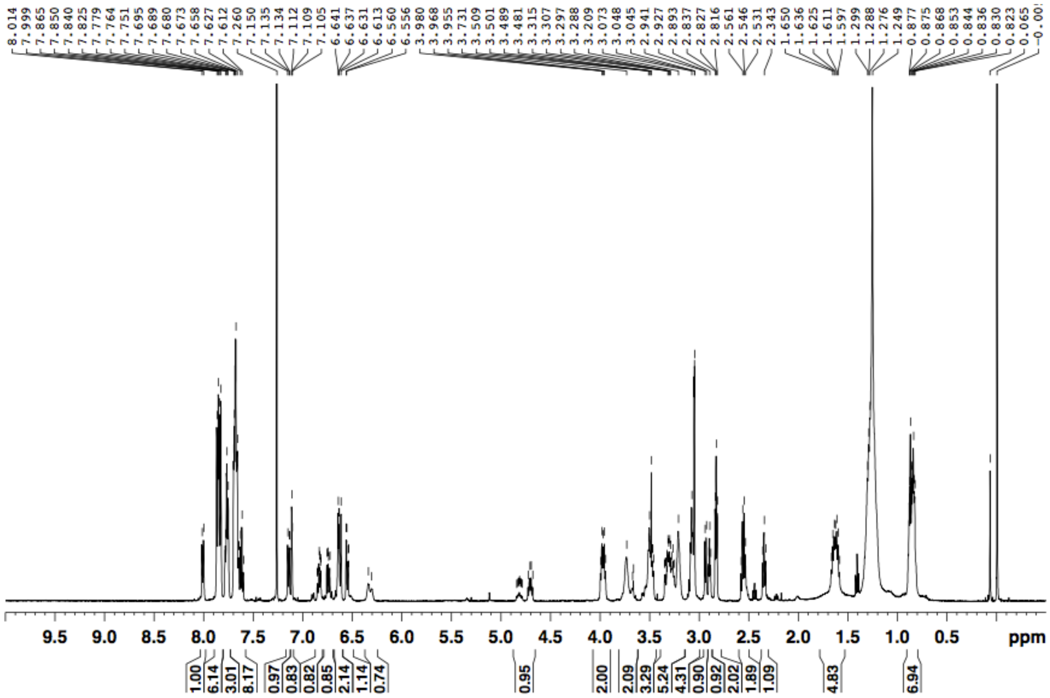

Supplementary Figure 36: ^{1}H-NMR of mitoDPP-2 (10).

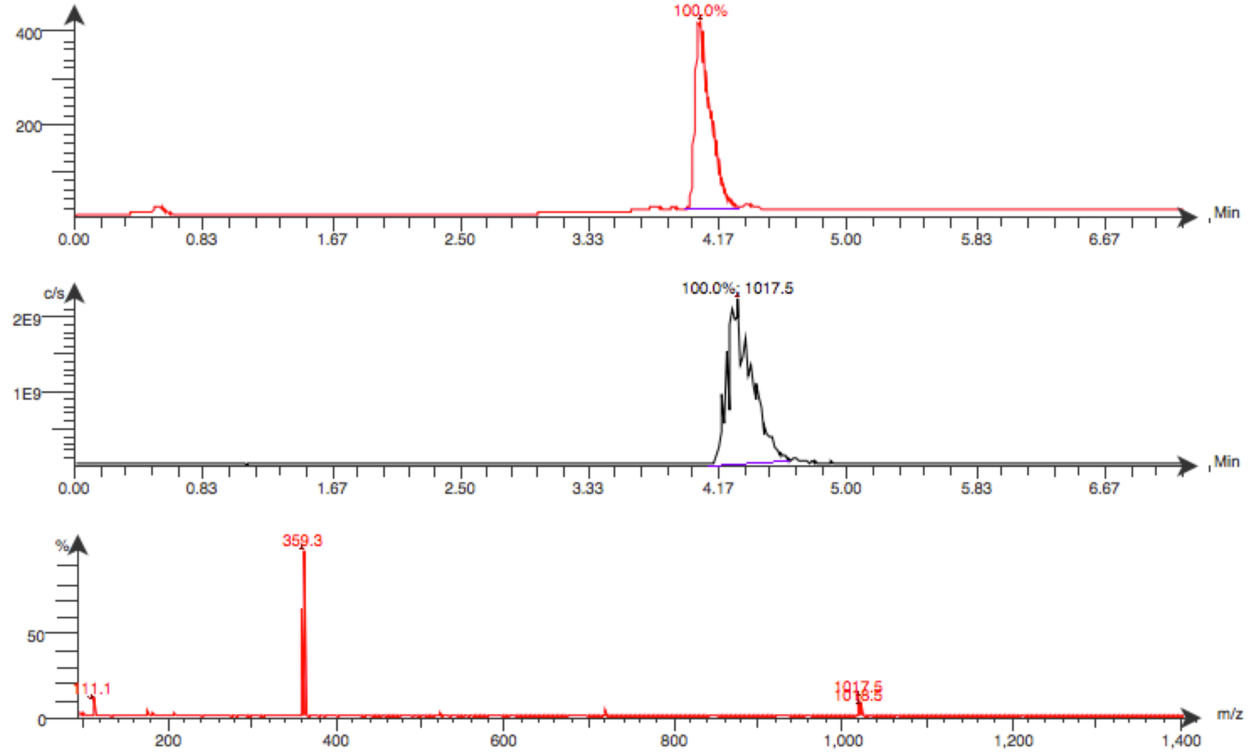

Supplementary Figure 37: LC/MS of mitoDPP-2 (10).

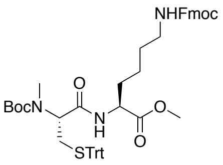

^{1}H-NMR (400 MHz; $CDCl_3$)

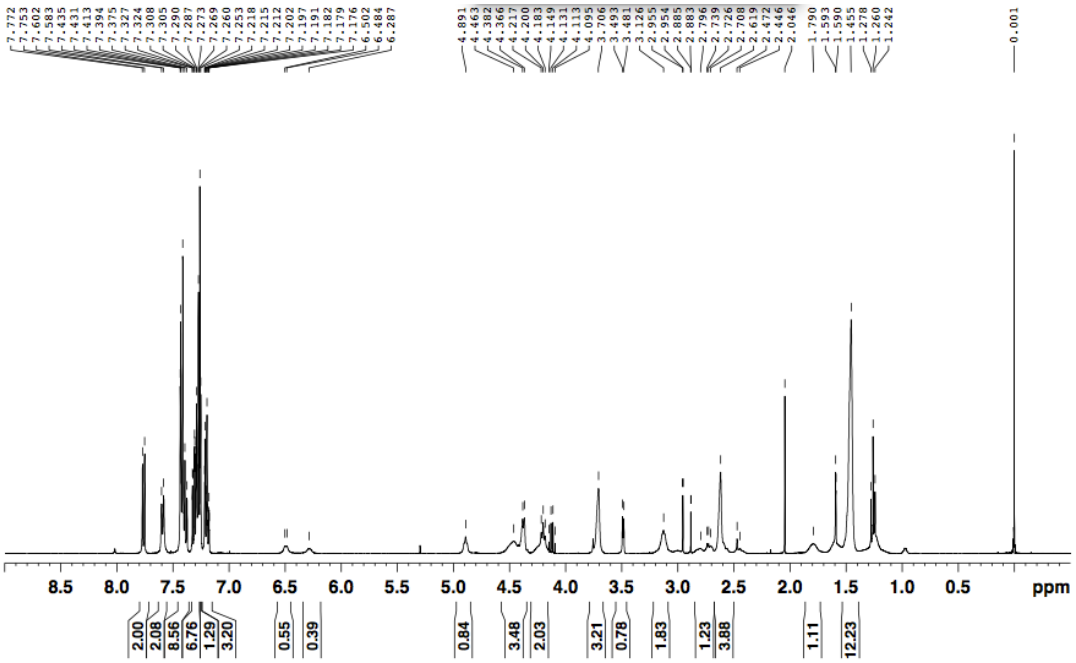

^{13}C-NMR (126 MHz; $CDCl_3$)

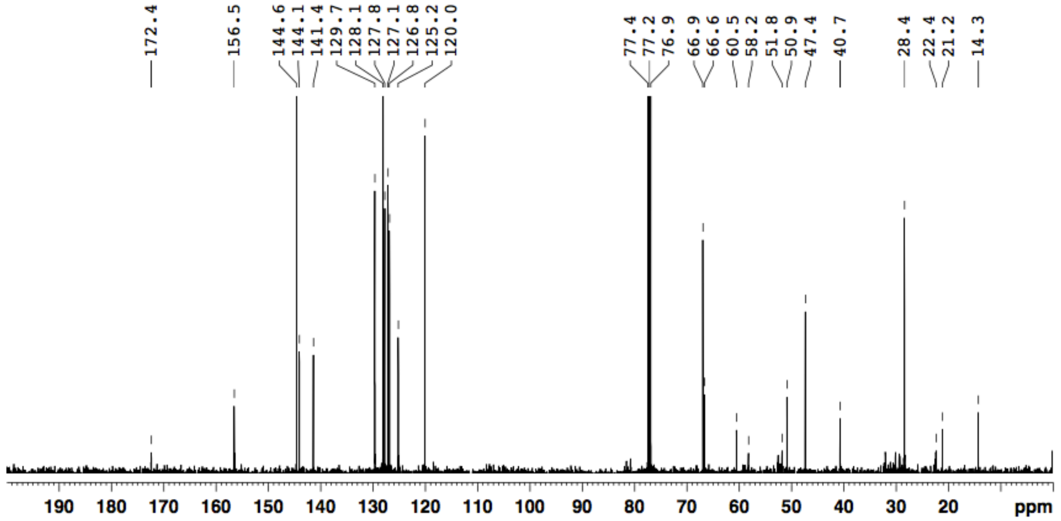

Supplementary Figure 38: ^{1}H-NMR and ^{13}C-NMR of 12.

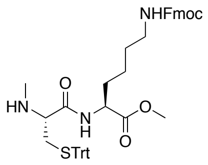

^{1}H-NMR (500 MHz; $CDCl_3$)

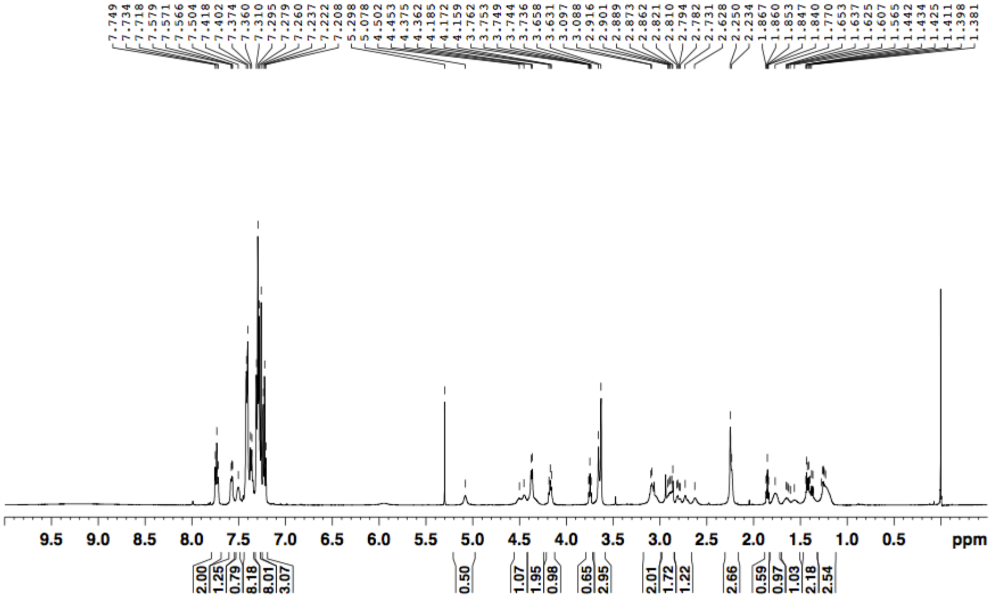

^{13}C-NMR (126 MHz; $CDCl_3$)

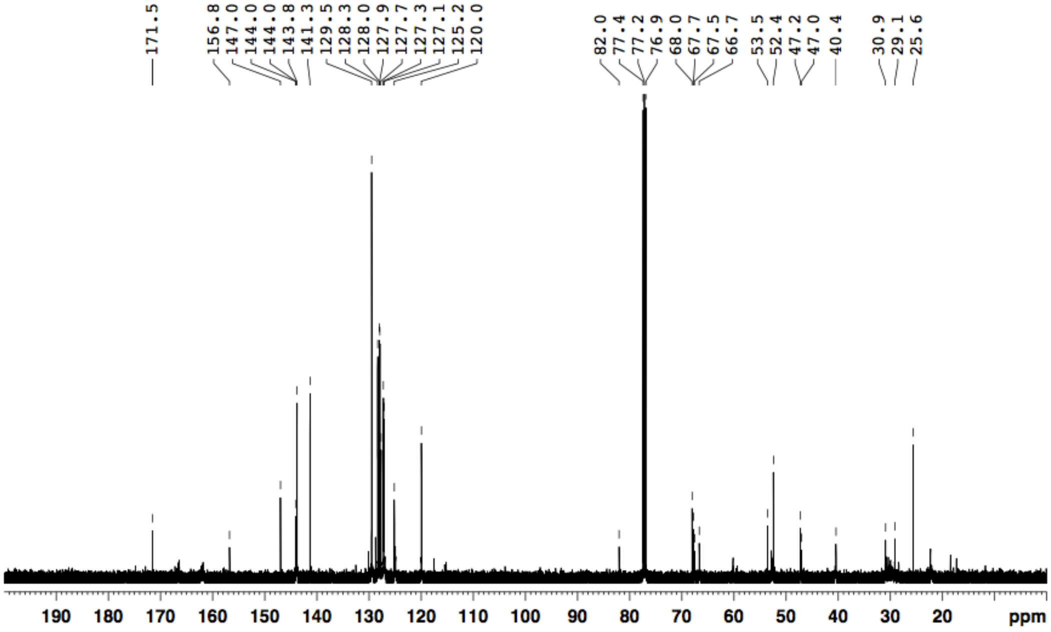

Supplementary Figure 39: ^{1}H-NMR and ^{13}C-NMR of 13.

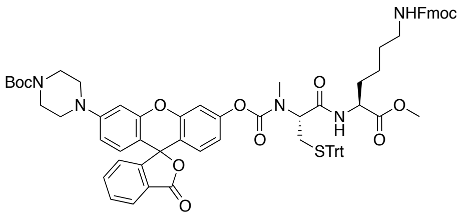

^{1}H-NMR (500 MHz; $CDCl_3$)

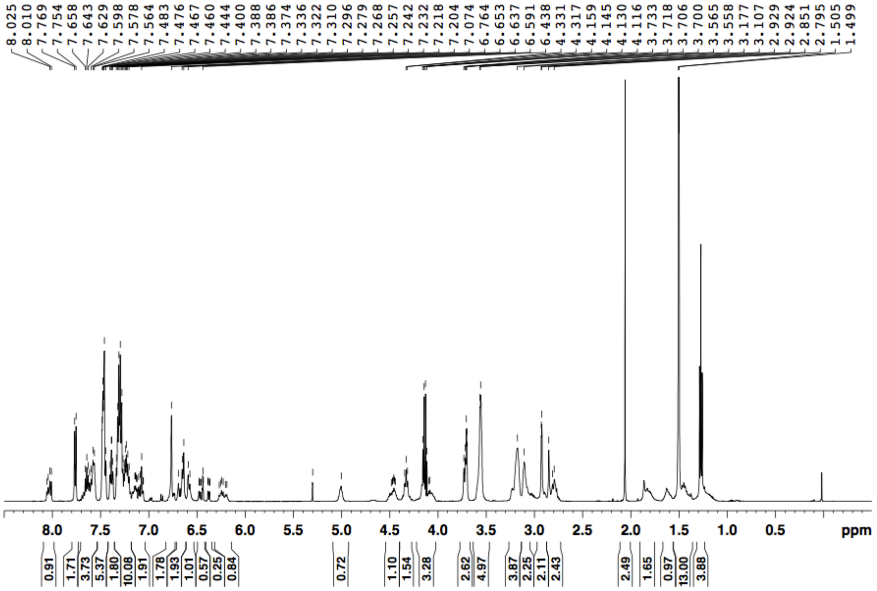

^{13}C-NMR (126 MHz; $CDCl_3$)

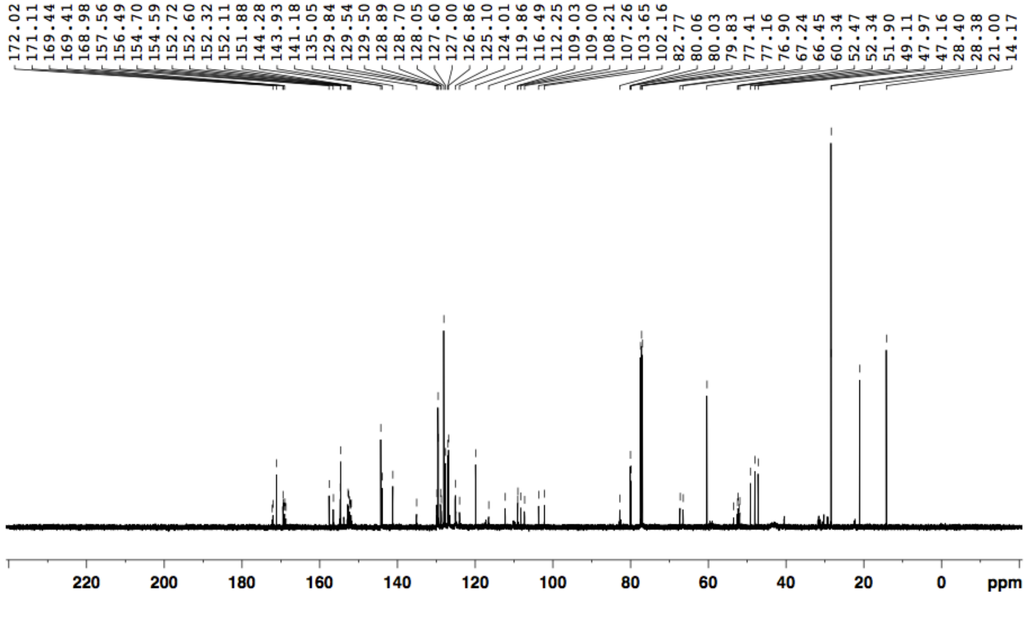

Supplementary Figure 40: ^{1}H-NMR and ^{13}C-NMR of 14.

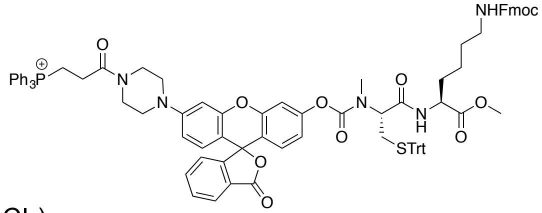

^1H-NMR (500 MHz; $CDCl_3$)

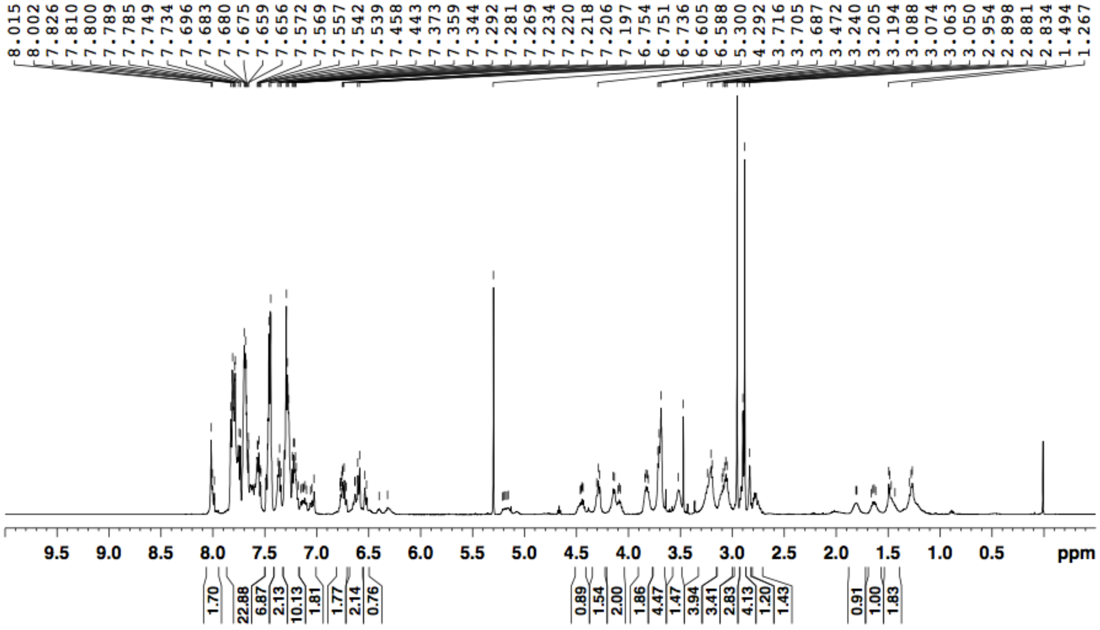

^{13}C-NMR (126 MHz; $CDCl_3$)

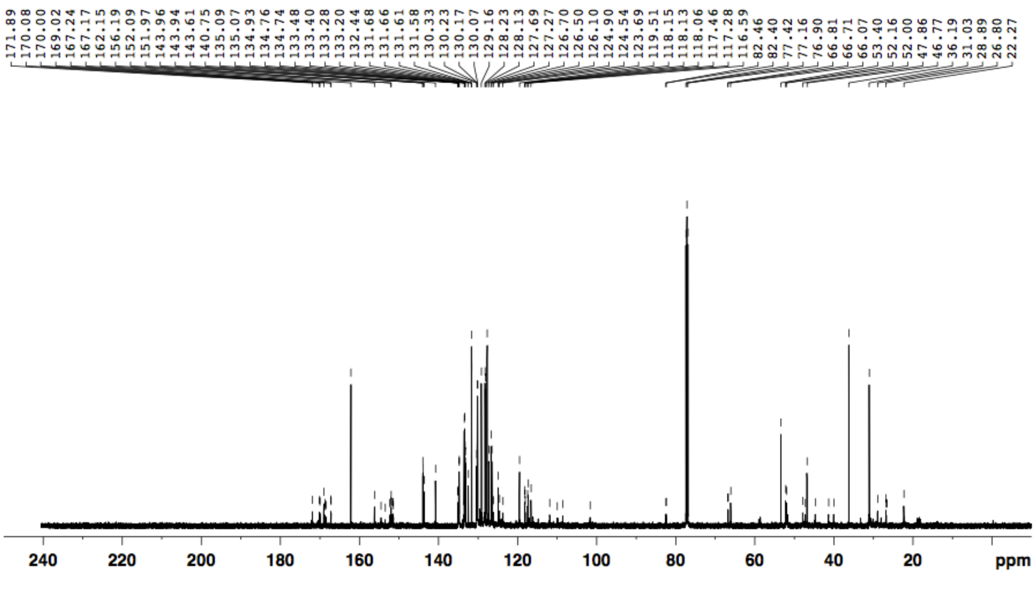

Supplementary Figure 41: ^{1}H-NMR and ^{13}C-NMR of 15.

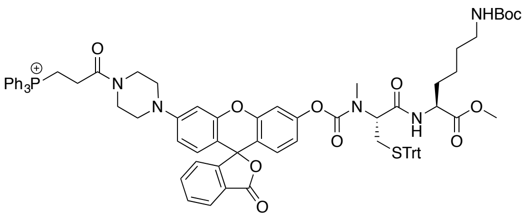

^{1}H-NMR (500 MHz; $CDCl_3$)

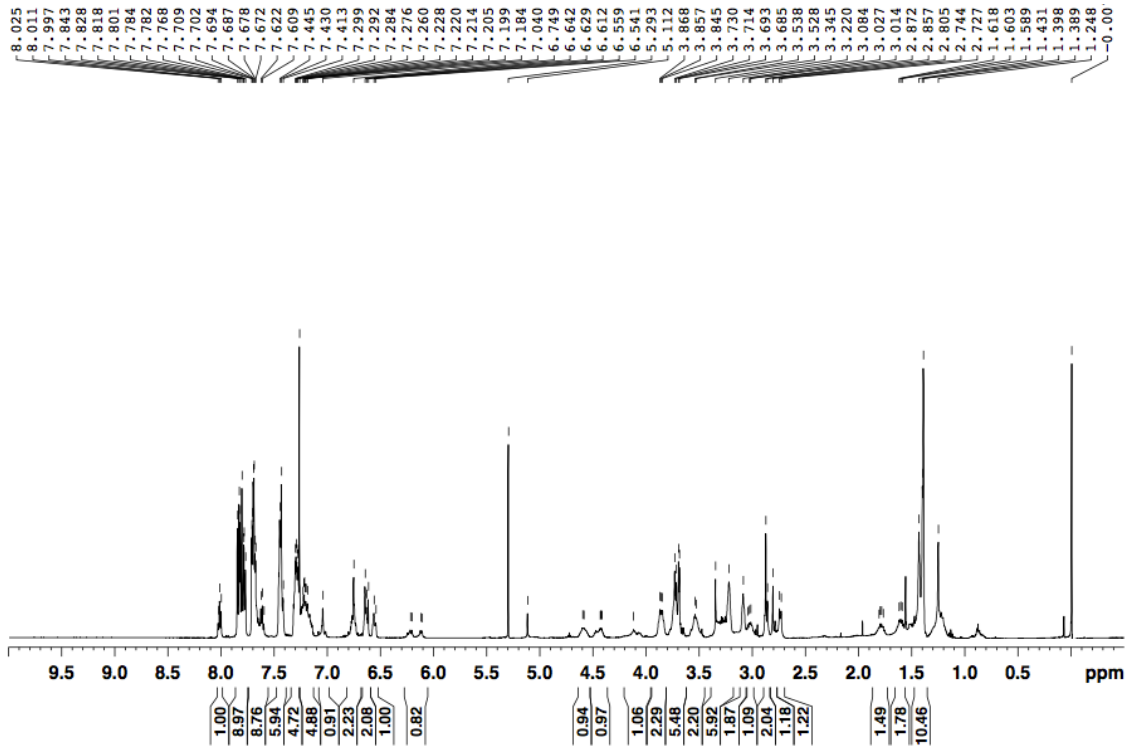

Supplementary Figure 42: ^{1}H-NMR of 16.

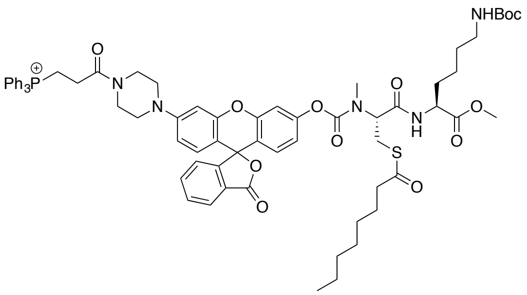

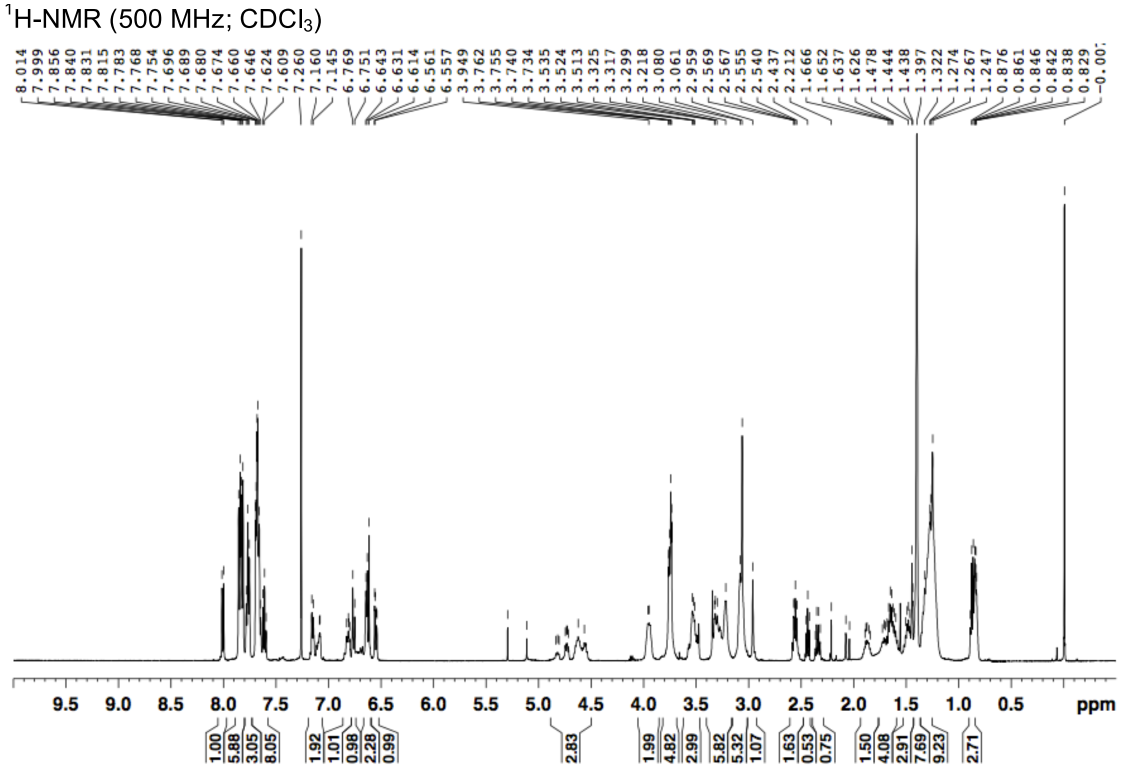

Supplementary Figure 43: ^{1}H-NMR of 17.

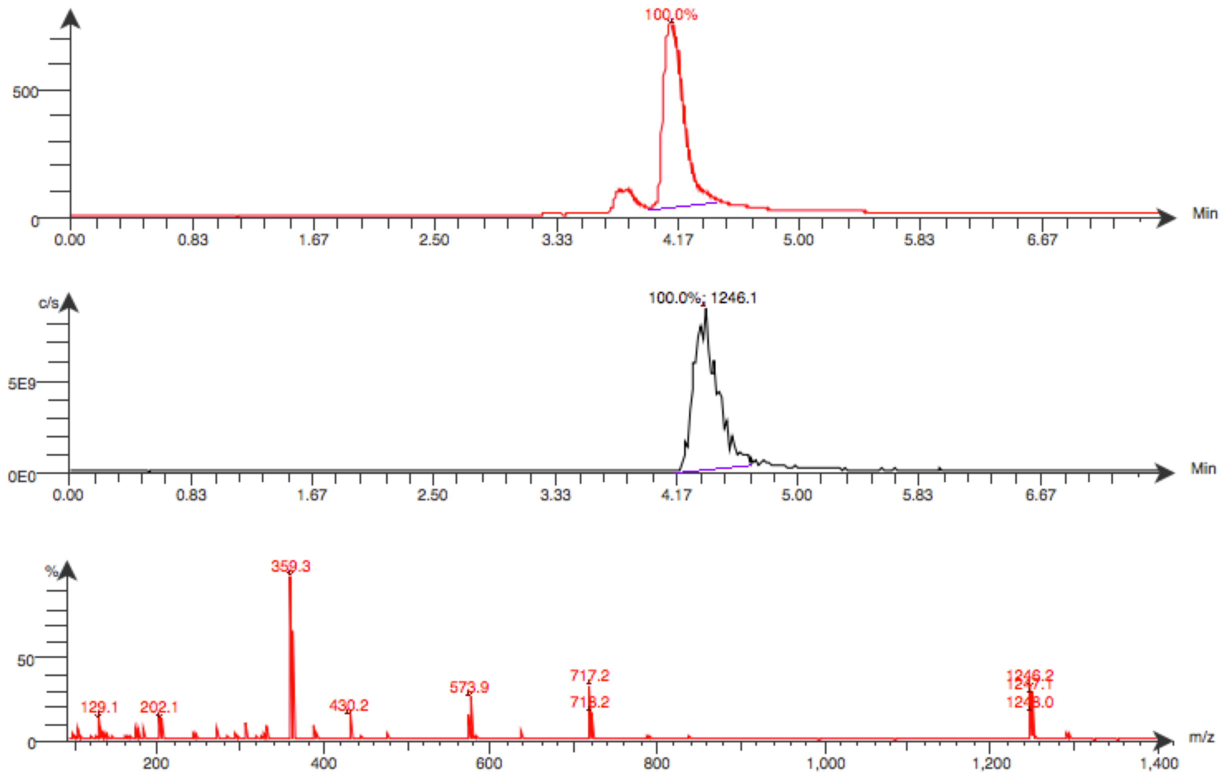

Supplementary Figure 44: LC/MS of 17.

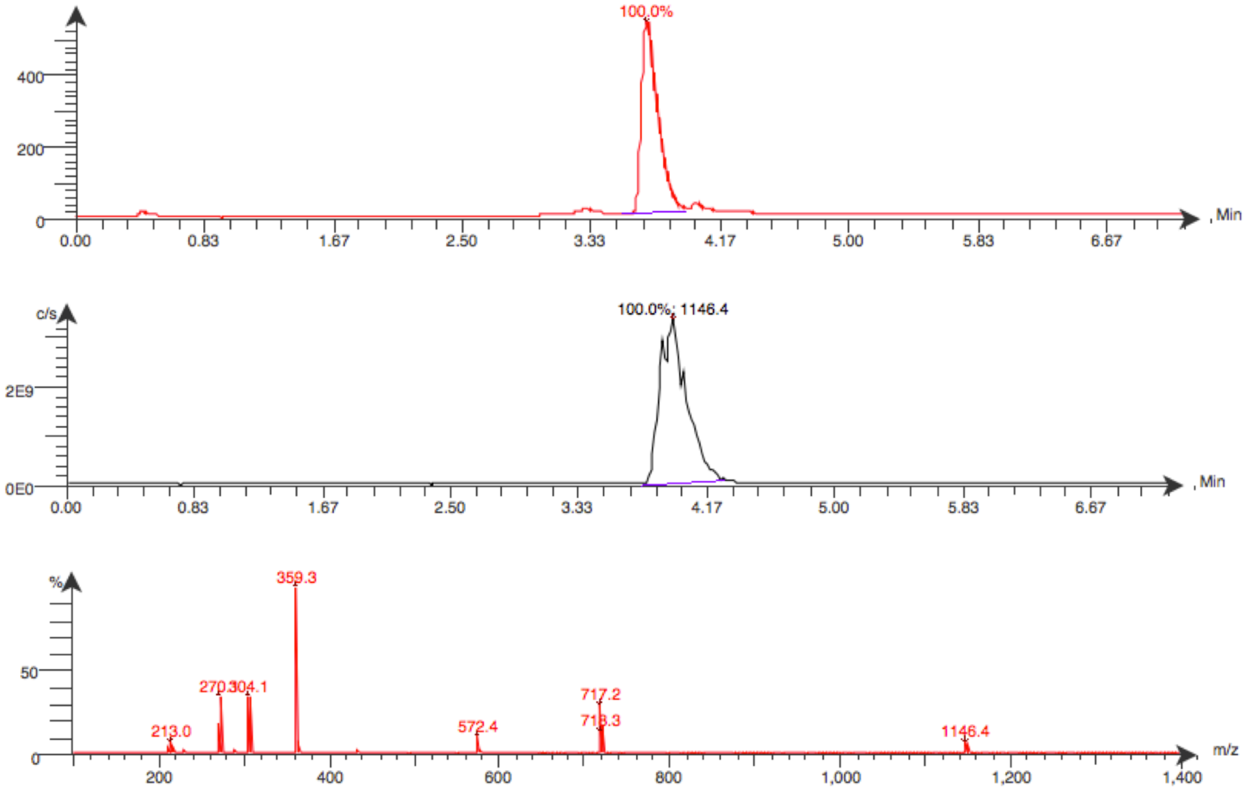

Supplementary Figure 45: LC/MS of mitoDPP-3 (18).

Supplementary Table 1: Enzymatic kinetic parameters for mitoDPP-2 and mitoDPP-3 with recombinant APT1.

	K_M (μM)	k_{cat} (s^{-1})	k_{cat}/K_M (10^5 s^{-1} M^{-1})
mitoDPP-2	3.01	0.64	2.1
mitoDPP-3	14.4	1.43	1.0

Supplementary Table 2: Sequences of RNAi used in this work.

**LYPLA1**	CGGTGGTGCTAATAGAGATAT
**LYPLA1**	CTATGCCTTCATGGTTTGATA
**LYPLA1**	CAGGAAATGATGGATGTCAA
**LYPLA2**	GGCTGCTTTCTTATCCATTTC
**LYPLA2**	GCAGCTGTGAAGGAATTTCTT
**LYPLAL1**	ATGAAAGGACTTAGCATAATG
**LYPLAL1**	CACTGTAGACAGTAGCTAATC
**ABHD17A**	AGATGAGCAGCTTCTACATTG
**ABHD17A**	GCACAACGACATCGAGCTCTA
**ABHD17B**	GGACTAGGATCACGGATTAAT
**ABHD17B**	GTGGATCTTGCTGCTCGATAT
**ABHD17C**	TATGAATGCGCAGCGGTAATT
**ABHD17C**	CATCAACTGTAACCATATAAA
**PPT1**	CAGATCCAGCTTGCAACTAAT
**PPT1**	GCACTTGCTAAGGATCCTAAA
**PPT2**	GCACTCCAACCGTACCCTTTA
**PPT2**	ACCTCATTGCTCCCATATTAT
**ACOT1**	TGATGGCTCTGGCTTACTATA
**ACOT1**	CCAGAGACAGGGCACTATATT
**ACOT2**	GCAGGTTGGTCAGATCATTAG
**ACOT2**	CGCTCCATCTGGAGTACTTTG
**ACOT4**	GCATTACATCGAGCCTCCTTA
**ACOT4**	CCAGGGATCATTGACATCTTT
**ACOT6**	CCTAAACTTGTCGATGATCTA
**ACOT6**	TGTTGGCATGGATGATCAAAG
**ACOT7**	AGGTGCCTCCTGTTGTGTATT
**ACOT7**	GCAATAAGTCCATGGAGATTG
**ACOT8**	GACCCTCATTGACCAGTATTT
**ACOT8**	CATTGGCGCTCAACCGAATTG
**ACOT9**	CCTGGTGGATAAGATTGATAT
**ACOT9**	GCCTGTAATCCCAGCACTTTA
**ACOT11**	TCTCGGCAAGTGGCTTCTATT
**ACOT11**	ATCACCAGGGCAACACCTTTG
**ACOT12**	ACATTTGGTGGCCAGATTATG
**ACOT12**	CCACAGTACATCAGAAGTGAA
**ACOT13**	CCACGTTAGTAGATAACATAT
**ACOT13**	TCTAATCATGTGTGATAATTG

Supplementary Table 3: Details of reduction in mRNA levels of targeted genes analyzed by rt-qPCR.

GENE	Primer 1	Primer 2	Fold reduction
APT1	CCCTTACCACACAGCAGAAA	CGTGGCACTGGAGAATAGAAA	1.65*
APT2	CAGGGTCCAGTTCAAGACATAC	TAGTTAGACAGGAGGCAGCA	1.41
PPT1	TGTAGATTCGGAGTGGTTTGG	CCTGCATTGTCCATTTCCTTTAG	2.27
PPT2	GGCCCTGATGATGGTGTTATTA	GATAAACCAGTTGCTCCTCCA	1.80
LYPLAL1	AGCCGAGAAGTTGTTACTGG	GCTCCACTGAGAATTAGGAGAC	1.74
ABHD17A	CGACACCAAGAAGACCTACTG	GATGATGAGCACGGGAGAC	1.80
ABHD17B	GTGATCCTCCCTCCTCAGTAT	GCATCACCTGGGAATCTTGATA	1.41
ABHD17C	GGGAATGAGAGCTGAATGTAGG	GTCCTGCATGAATGTCAGAAATG	2.43
ACOT1	CTTGGTGGGCAGTCCTATTATC	CCCAAGTGTTTGTGGAAGAAAG	3.10*
ACOT11	GATGACAAGTTCCTCTCCTTCC	GTAGTGCTTGTCCCACTCTG	2.20*

**mean from two biological replicates*

GENE	Control shRNA (target : GAPDH)	Target shRNA (target : GAPDH)
APT1	25.84 : 20.07	26.38 : 19.88
APT2	26.62 : 18.87	27.53 : 18.87
PPT1	24.25 : 19.83	24.93 : 19.33
PPT2	26.11 : 19.19	26.76 : 18.97
LYPLAL1	26.80 : 18.87	28.03 : 19.30
ABHD17A	26.83 : 19.29	27.21 : 18.82
ABHD17B	30.47 : 19.29	30.85 : 19.17
ABHD17C	25.51 : 19.29	27.17 : 19.67
ACOT1	25.89 : 20.78	27.39 : 20.67
ACOT11	30.74 : 19.62	32.37 : 20.13

**C_t values shown for each identified gene relative to GAPDH*

Supplementary Table 4: Details for confocal imaging with mitoDPP-2 and mitoDPP-3.

a) mitoDPP-2

	Hoechst (405 nm/412-452 nm)	MitoDDP-2 (514 nm/520-590nm)	MitoTracker (633 nm/638-722 nm)
**HEK293T Cells**			
laser intensity	44%	63%	3%
Gain	1036	100	100
**MCF7 Cells**			
laser intensity	44%	100%	3%
Gain	1036	340	100
**A549 Cells**			
laser intensity	44%	15%	3%
Gain	1036	340	100

b) mitoDPP-3

	Hoechst (405 nm/412-452 nm)	MitoDDP-3 (514 nm/520-590nm)	MitoTracker (633 nm/638-722 nm)
**HEK293T Cells**			
laser intensity	60%	48%	3%
Gain	1036	100	100

SUPPLEMENTARY METHODS: SYNTHETIC METHODS

Synthesis of 2. Synthesis adapted from literature[2]. A mixture of **1** (845.8 mg, 1.0 eq, 4.23 mmol) and triphenylphosphine (1.109 g, 1.0 eq, 4.23 mmol) in acetonitrile (10 mL) was refluxed for 6 h. The reaction mixture was then evaporated, and subsequent washing with EtOAc and drying on vacuum afforded **2** (1.388 g), which was used without further purification. **HRA-MS(+)**: Calculated for $C_{21}H_{20}O_2P^+$ [M^+] 335.1201; found 335.1215.

Synthesis of 6. Adapted from literature[3]. To a mixture of **3** (2.143 g, 1.0 eq, 12.02 mmol) and **4** (3.053 g, 1.0 eq, 11.82 mmol) in pressure flask added TFA (5 mL), and the resulting reaction mixture was stirred at 95 ^oC for 9 h. The reaction mixture was diluted three times with 30 mL of DCM and followed by rotatory evaporation to remove residual TFA. To the resultant crude material Et_3N (7.2 mL, 5 eq, 51.7 mmol) and MeOH (25 mL) were added. After stirring for 5 min, Boc_2O (2.106 g, 0.93 eq, 9.6 mmol) was added. After stirring the resultant reaction mixture overnight at room temperature, the MeOH was removed by rotary evaporation. To the crude mixture, K_2CO_3 (4.287 g, 3.0 eq, 31.0 mmol), pivalic anhydride (2.1 mL, 1.0 eq, 10.3 mmol) and DMF (15 mL) were added and the reaction stirred for 1 h. The reaction mixture was diluted with 30 mL DCM, filtered, and evaporated. The crude product was purified by column chromatography (Silica; 0-20% EtOAc:DCM) to yield **5**. The combined fractions of **5** were evaporated and dissolved in MeOH (60 mL), to which 3 M NaOH (30 mL) was added, and reaction mixture stirred for 30 min at room temperature. The reaction mixture was then evaporated by rotary evaporation, the crude product was diluted by DCM (50 mL), and then washed by aq. HCl (pH ~ 3-4). The aqueous layer was further extracted by DCM 2 x 30 mL, the combined organic layers were dried over Na_2SO_4, and then evaporated to give a crude product. Purification by column chromatography (Silica; 0-5% MeOH:DCM) yielded **6** (3.892 g, 65%). **R_f**: 0.41 (Silica; 5% MeOH:DCM). **^{1}H-NMR** (500 MHz; $CDCl_3$): δ 9.04-8.92 (b, 1H), 8.02 (dd, *J* = 6.4, 1.9 Hz, 1H), 7.61-7.55 (m, 2H), 7.10-7.09 (m, 1H), 6.73 (d, *J* = 8.8 Hz, 1H), 6.67-6.64 (m, 2H), 6.60-6.52 (m, 3H), 3.51 (d, *J* = 4.7 Hz, 4H), 3.21 (s, 4H), 1.46 (s, 9H). **^{13}C-NMR** (126 MHz; $CDCl_3$):170.3, 162.1, 154.9, 153.8, 153.5, 148.6, 134.0, 129.8, 129.6, 129.5, 129.2, 126.2, 125.2, 114.3, 112.5, 111.3, 110.7, 103.1. 80.6, 53.5, 52.4, 49.2, 47.7, 28.5, 27.2, 27.1. **HRA-MS(+)**: Calculated for $C_{29}H_{28}N_2O_6$ [M^+] 500.1947; found 500.1953.

Synthesis of 8. To a solution of **6** (306.4 mg, 0.612 mmol, 1.0 eq) in dry THF (7 mL) Et_3N (0.8 M, 0.77 mL, 0.613 mmol, 1.0 eq) was added. The resultant solution was added dropwise over 15 min to a solution of triphosgene (181.6 mg, 0.612 mmol, 1.0 eq) in dry THF (5 mL) on an ice bath. After 5 min, the ice bath was replaced by a 40 ^oC water bath and N_2 flushed through the reaction mixture to evaporate the solvent completely. Then, the 40 ^oC water bath was removed and the

crude product was resuspended in dry THF (4 mL). To this reaction mixture, **7**[4] (0.32 M, 1.88 mL, 0.609 mmol, 1.0 eq), followed by Et_3N (0.8 M, 0.77 mL, 0.613 mmol, 1.0 eq), were added dropwise. After 15 min of stirring, the reaction mixture was quenched by five drops of 1M HCl and the solvent evaporated by rotary evaporation. The crude reaction was purified by column chromatography (Silica; 5-25% EtOAc:DCM) to yield **8** (259.8 mg; 46%). **R_f**: 0.39 (Silica; 15% EtOAc:DCM). **^{1}H-NMR** (500 MHz; $CDCl_3$): δ 8.05 (t, *J* = 6.5 Hz, 1H), 7.70-7.62 (m, 2H), 7.50-7.45 (m, 6H), 7.33-7.09 (m, 11H), 6.84-6.63 (m, 5H), 6.15-6.07 (m, 1H), 4.35 (m, 4.9 Hz, 1H), 3.61 (t, *J* = 4.8 Hz, 4H), 3.25 (d, *J* = 4.5 Hz, 4H), 2.90 (d, *J* = 0.8 Hz, 2H), 2.83-2.68 (m, 5H), 1.53 (s, 10H). **^{13}C-NMR** (126 MHz; $CDCl_3$): δ 169.4, 169.1, 169.1, 155.0, 154.6, 153.0, 153.0, 152.9, 152.8, 152.4, 152.2, 151.9, 151.9, 144.4, 135.1, 129.8, 129.6, 129.5, 128.9, 128.8, 128.0, 126.8, 126.6, 125.0, 124.0, 123.9, 117.3, 117.3, 116.5, 116.4, 112.3, 110.3, 110.2, 109.1, 102.2, 82.8, 82.7, 80.1, 67.2, 67.1, 67.1, 67.0, 58.5, 48.1, 31.3, 30.8, 30.7, 30.3, 28.4, 26.3, 26.1. **HRA-MS(+)**: Calculated for $C_{54}H_{52}N_4O_8S$ [M^+] 916.3506; found 916.3508.

Synthesis of 9. A solution of **8** (143.3 mg, 1.0 eq, 0.156 mmol) in 20% TFA:DCM (5 mL) was stirred for 45 min at room temperature, followed by dilution with DCM (3x 20 mL) and evaporation by rotary evaporation. The obtained crude was resuspended in dry DMF (4 mL) and **2** (57.6 mg, 0.172 mmol, 1.1 eq), HOBt (80%, 38.9 mg, 0.229 mmol, 1.5 eq), and EDC.HCl (40.2 mg, 0.210 mmol, 1.4 eq) were added, the reaction mixture was stirred at room temperature for 5 min, and then Et_3N (43.6 µL, 0.313 mmol, 2 eq) was added. After 70 min, the reaction mixture was evaporated by rotary evaporation and the resultant crude product was purified by column chromatography (Silica; 5-10% MeOH:DCM) to yield **9** (155.0 mg, 87%). **R_f**: 0.44 (Silica; 10% MeOH:DCM). **^{1}H-NMR** (500 MHz; $CDCl_3$): δ 8.00 (d, *J* = 7.6 Hz, 1H), 7.81-7.76 (m, 8H), 7.70-7.63 (m, 8H), 7.46-7.42 (m, 6H), 7.32-7.09 (m, 10H), 6.82 (dtd, *J* = 15.8, 8.0, 2.2 Hz, 1H), 6.71-6.55 (m, 4H), 6.41 (dd, *J* = 10.1, 4.9 Hz, 1H), 4.41-4.32 (m, 1H), 3.81 (dt, *J* = 12.4, 6.3 Hz, 2H), 3.63 (s, 2H), 3.51 (s, 2H), 3.19 (s, 4H), 3.07 (s, 2H), 2.95 (s, 1H), 2.88-2.82 (m, 3H), 2.77-2.69 (m, 4H), 2.25 (br, 1H), 1.28 (s, 1H). **^{13}C-NMR** (126 MHz; $CDCl_3$): δ 169.4, 167.7, 167.6, 154.9, 154.87, 152.8, 152.4, 152.3, 152.1, 151.8, 151.8, 144.3, 135.2, 135.0, 135.0, 133.8, 133.8, 130.4, 130.3, 129.8, 129.5, 129.5, 128.7, 127.9, 126.8, 126.4, 124.9, 118.6, 117.9, 116.2, 122.2, 108.9, 102.0, 82.8, 82.8, 67.0, 66.9, 58.5, 58.5, 53.5, 48.2, 47.5, 45.0, 41.6, 30.9, 30.8, 30.4, 27.2, 26.3, 26.1, 19.2, 18.7. **HRA-MS(+)**: Calculated for $C_{70}H_{62}N_4O_7PS^+$ [M^+] 1133.4077; found 1133.4071.

Synthesis of mitoDPP-2 (10). To a solution of **9** (106.3 mg, 93.7 µmol, 1.0 eq) in MeOH (20 mL), I_2 (55.4 mM in MeOH; 6.8 mL, 376.7 µmol, 4.0 eq) was added dropwise over 30 min. The reaction mixture was quenched with a solution of 0.2 M sodium citrate and 0.2 M sodium ascorbate at pH ~3-4 until the yellow color disappeared. The quenched reaction mix was diluted with DCM (25

mL) and washed with brine (25 mL). The aqueous layer was washed again with DCM (25 mL). The combined organic layers were dried over Na_2SO_4 and evaporated by rotary evaporation. The resultant crude containing the disulfide product was resuspended in a mixture of 1:1 MeOH:H_2O (4 mL) and TCEP.HCl (138.4 mg, 482.8 µL, 5.0 eq) was added. The reaction mixture was stirred at room temperature for 30 min, followed by the additon of octanoyl anhydride (2 mL) and subsequent additon of Et_3N (195 µL, 1.40 mmol, 15.0 eq) in portions of 6.5 µL. The resulting reaction mixture was diluted with DCM (25 mL) and washed with brine (20 mL). The aqueous layer was washed again with DCM (20 mL) and the combined organic layers were dried over Na_2SO_4 and evporated by rotary evaporation. Purification by column chromatography (Silica; 0-10% MeOH:DCM) afforded **mitoDPP-2** (**10**; 23.2 mg; 24%). Purity was assayed by LC/MS. **^{1}H-NMR** (500 MHz; $CDCl_3$): δ 8.01 (d, *J* = 7.8 Hz, 1H), 7.87-7.82 (m, 6H), 7.78-7.75 (m, 3H), 7.70-7.60 (m, 8H), 7.14 (dd, *J* = 7.5, 0.8 Hz, 1H), 7.11 (t, *J* = 2.2 Hz, 1H), 6.83 (td, *J* = 7.8, 2.2 Hz, 1H), 6.74 (dd, *J* = 8.7, 6.2 Hz, 1H), 6.64-6.61 (m, 2H), 6.55 (dd, *J* = 8.8, 2.2 Hz, 1H), 6.32 (d, *J* = 18.6 Hz, 1H), 4.85-4.68 (m, 1H), 3.97 (dt, *J* = 12.4, 6.3 Hz, 2H), 3.73-3.66 (m, 2H), 3.54-3.46 (m, 3H), 3.35-3.25 (m, 3H), 3.21 (s, 2H), 3.10-3.04 (m, 4H), 2.93 (d, *J* = 6.9 Hz, 1H), 2.89 (t, *J* = 5.0 Hz, 1H), 2.83 (t, *J* = 5.3 Hz, 2H), 2.55 (q, *J* = 7.5 Hz, 2H), 2.34 (t, *J* = 7.5 Hz, 1H), 1.62 (dq, *J* = 20.2, 7.0 Hz, 5H), 0.88-0.82 (m, 7H). **HRA-MS(+):** Calculated for $C_{59}H_{62}N_4O_8PS^+$ [M^+] 1017.4026; found 1017.4051.

Synthesis of 12. DIPEA (215 µL; 1.23 mmol; 1.1 eq) was added to a mixture of **11**[4] (530.0 mg; 1.11 mmol; 1.0 eq), EDC.HCl (282.4 mg; 1.47 mmol; 1.3 eq), HOBt (80%, 241.3 mg; 1.43 mmol; 1.3 eq), and Fmoc-Lysine(OMe).HCl (472.9 mg; 1.13 mmol; 1.0 eq) in 4 mL DMF. The resulting reaction mixture was stirred at room temperature for 20 min. The reaction mixture evaporated by rotary evaporation and purified by column chromatography (Silica; 4-15% EtOAc:DCM) to yield **12** (838.6 mg; 90%). **R_f**: 0.16 (Silica; 5% EtOAc:DCM). **^{1}H-NMR** (400 MHz; $CDCl_3$): δ 7.76 (d, *J* = 7.5 Hz, 2H), 7.59 (d, *J* = 7.4 Hz, 2H), 7.44-7.38 (m, 9H), 7.33-7.27 (m, 7H), 7.25 (s, 1H), 7.22-7.18 (m, 3H), 6.49 (br, 1H), 4.89 (s, 1H), 4.46-4.37 (m, 3H), 4.20 (t, *J* = 6.8 Hz, 2H), 3.71 (s, 3H), 3.49 (d, *J* = 5.0 Hz, 1H), 3.13-3.11 (s, 2H), 2.80-2.71 (m, 1H), 2.62 (s, 4H), 1.79 (s, 1H), 1.45 (s, 12H). **^{13}C-NMR** (126 MHz; $CDCl_3$): δ 172.4, 156.5, 144.6, 144.1, 141.4, 129.7, 128.1, 127.8, 127.1, 126.8, 125.2, 120.0, 66.9, 66.6, 60.5, 58.2, 51.8, 50.9, 47.4, 40.7, 28.4, 22.4, 21.2, 14.3. **HRA-MS(+):** Calculated for $C_{50}H_{55}N_3O_7S$ [M^+] 841.3761; found 841.3737.

Synthesis of 13. **12** (786.2 mg; 0.934 mmol; 1.0 eq) was added to 10 mL of 20% TFA:DCM. The reaction mixture was stirred for 30 min and then diluted with 20 mL DCM followed by rotary evaporation. This procedure was repeated three times with 20 mL DCM. Purification by column chromatography (Silica; 0-5% MeOH in 1:1 EtOAc:DCM) yielded **13** (675.9 mg; 97%). **R_f**: 0.45

(Silica; 1% MeOH in 1:1 EtOAc:DCM). **^{1}H-NMR** (500 MHz; $CDCl_3$): δ 7.73 (t, *J* = 7.8 Hz, 2H), 7.59-7.57 (m, 1H), 7.51-7.49 (m, 1H), 7.42-7.35 (m, 8H), 7.31-7.28 (m, 8H), 7.22 (t, *J* = 7.3 Hz, 3H), 5.08 (br, 1H), 4.47 (d, *J* = 25.0 Hz, 1H), 4.38-4.36 (m, 2H), 4.17 (t, *J* = 6.6 Hz, 1H), 3.76-3.74 (m, 1H), 3.64 (d, *J* = 13.9 Hz, 3H), 3.09 (br, 2H), 2.94-2.86 (m, 2H), 2.82-2.62 (m, 1H), 2.25 (s, 3H), 1.87-1.84 (m, 1H), 1.77 (br, 1H), 1.68-1.55 (m, 1H), 1.44-1.37 (m, 2H), 1.26-1.21 (m, 2H). **^{13}C-NMR** (126 MHz; $CDCl_3$): δ 171.5, 156.8, 147.0, 144.0, 144.0, 143.8, 141.3, 129.5, 128.3, 128.0, 127.9, 127.7, 127.3, 127.1, 125.2, 120.0, 82.0, 68.0, 67.7, 67.5,66.7, 53.5, 52.4, 47.2, 47.0, 40.4, 30.9, 29.1, 25.6. **HRA-MS(+)**: Calculated for $C_{45}H_{47}N_3O_5S$ [M^+] 741.3236; found 741.3232.

Synthesis of 14. To a solution of **6** (318.9 mg, 0.632 mmol, 1.0 eq) in dry THF (10 mL) Et_3N (0.90 M, 0.7 mL, 0.63 mmol, 1.0 eq) was added. The solution was added dropwise over 15 min to a solution of triphosgene (178.2 mg, 0.60 mmol, 1 eq) in dry THF (6 mL) on an ice bath. After 5 min, the ice bath was replaced by a 40 ^{o}C water bath and N_2 flushed through the reaction mixture to evaporate the solvent completely. Then, the 40 ^{o}C water bath was removed and crude mixture was resuspended in dry THF (5 mL). To this reaction mixture, **13** (0.1 M, 4.5 mL, 0.472 mmol, 0.75 eq), followed by Et_3N (0.90 M; 0.53 mL, 0.47 mmol, 0.75 eq), were added dropwise. After 5 min of stirring the reaction mixture was quenched by five drops of 1M HCl and the solvent evaporated by rotary evaporation. Purification by column chromatography (Silica; 5-35% EtOAc:DCM) yielded **14** (330.4 mg; 55%). **R_f**: 0.34 (Silica; 15% EtOAc:DCM). **^{1}H-NMR** (500 MHz; $CDCl_3$): δ 8.03 (dd, *J* = 16.1, 7.3 Hz, 1H), 7.76 (d, *J* = 7.5 Hz, 2H), 7.70-7.53 (m, 4H), 7.46 (dt, *J* = 4.8, 2.8 Hz, 5H), 7.40-7.37 (m, 2H), 7.34-7.19 (m, 10H), 7.17-7.06 (m, 2H), 6.76 (s, 2H), 6.92-6.64 (m, 2H), 6.59-6.57 (m, 1H), 6.48-6.43 (m, 1H), 6.27-6.19 (m, 1H), 5.00 (t, *J* = 5.6 Hz, 1H), 4.50-4.44 (m, 1H), 4.37-4.29 (m, 2H), 4.10-4.05 (m, 1H), 3.73-3.70 (m, 3H), 3.56 (d, *J* = 4.1 Hz, 5H), 3.18 (br, 4H), 3.10 (m, 3H), 2.93 (d, *J* = 2.7 Hz, 2H), 2.85-2.75 (m, 2H), 1.86-1.79 (br, 2H), 1.63-1.58 (m, 1H), 1.51-1.44 (m, 13H). **^{13}C-NMR** (126 MHz; $CDCl_3$): 172.0, 171.1, 169.4, 169.4, 169.0, 157.6, 156.5, 154.7, 154.6, 152.7, 152.6, 152.3, 152.1, 151.9, 144.3, 143.9, 141.2, 135.0, 129.8, 129.5, 129.5, 128.9, 128.7, 128.0, 127.6, 127.0, 126.9, 125.1, 124.0, 119.9, 112.2, 109.0, 109.0, 108.2, 107.3, 103.7, 102.2, 82.8, 80.0, 80.0, 79.8, 67.2, 66.4, 60.3, 52.5, 52.3, 51.9, 49.9, 48.0, 47.2, 28.4, 28.4, 21.0, 14.2. **HRA-MS(+)**: Calculated for $C_{75}H_{73}N_5O_{12}S$ [M^+] 1267.4976; found 1267.4961.

Synthesis of 15. A solution of **14** (325.4 mg, 1.0 eq, 0.256 mmol) in 20% TFA:DCM (5 mL) was stirred for 30 min at room temperature, followed by dilution with DCM (3x 20 mL) and evaporation by rotatory evaporation. The resulting crude was suspended in dry DMF (5 mL) and **2** (172.1 mg, 0.513 mmol, 2.0 eq), HOBt (69.3 mg, 0.410 mmol, 1.6 eq), EDC.HCl (98.3 mg, 0.513 mmol, 2.0 eq) and Et_3N (180.0 µL, 1.29 mmol, 5.0 eq) were added. The reaction mix was stirred at room

temperature for 45 min. The reaction was evaporated by rotary evaporation, followed by purification by column chromatography (Silica; 1-7% MeOH:DCM) to yield **15** (328.5 mg, 86%). **R$_f$**: 0.38 (Silica; 5% MeOH:DCM). **^{1}H-NMR** (500 MHz; $CDCl_3$): δ 8.01 (d, *J* = 14.4 Hz, 2H), 7.83-7.54 (m, 23H), 7.49-7.44 (m, 7H), 7.37-7.34 (m, 2H), 7.31-7.18 (m, 10H), 7.15-7.03 (m, 2H), 6.78-6.71 (m, 2H), 6.65-6.59 (m, 2H), 6.53 (d, *J* = 9.0 Hz, 1H), 4.50-4.42 (m, 1H), 4.29 (t, *J* = 7.3 Hz, 1H), 4.15-4.05 (m, 2H), 3.84-3.81 (m, 2H), 3.70 (dd, *J* = 12.8, 4.0 Hz, 4H), 3.53-3.51 (br, 2H), 3.25-3.19 (m, 4H), 3.12-3.02 (m, 3H), 2.95 (d, *J* = 0.4 Hz, 3H), 2.90-2.88 (m, 4H), 2.83 (d, *J* = 1.0 Hz, 1H), 2.80-2.74 (m, 1H), 1.83-1.79 (br, 1H), 1.68-1.60 (m, 1H), 1.49-1.43 (m, 2H). **^{13}C-NMR** (126 MHz; $CDCl_3$): δ 171.9, 170.1, 170.0, 169.0, 167.2, 167.2, 162.2, 156.2, 152.1, 152.0, 144.0, 143.9, 143.6, 140.8, 135.1, 135.1, 134.9, 134.8, 134.7, 133.5, 133.4, 133.3, 133.2, 132.4, 131.7, 131.7, 131.6, 131.6, 130.3, 130.2, 130.2, 130.1, 129.2, 128.2, 128.1, 127.7, 127.3, 126.7, 126.5, 126.1, 124.9, 124.5, 123.7, 119.5, 118.6, 118.1, 118.1, 117.5, 117.3, 116.6, 82.5, 82.4, 66.8, 66.7, 66.1, 53.4, 52.2, 52.0, 47.9, 46.8, 36.2, 31.0, 28.9, 26.8, 22.3. **HRA-MS(+)**: Calculated for $C_{91}H_{83}N_5O_{11}PS^+$ [M^+] 1484.5547; found 1484.5486.

Synthesis of 16. A solution of **15** (48.3 mg, 1.0 eq, 32.5 µmol) in 20% Piperidine:DCM (2 mL) was stirred for 30 min at room temperature. The reaction mixture was then diluted with DCM (20 mL) and washed with brine (pH ~ 3-4). The aqueous layer was washed again with DCM (15 mL). The combined organic layers were dried with Na_2SO_4 and evaporated by rotary evaporation. The resultant crude was resuspended in MeOH (2 mL), and Boc_2O (350.0 mg, 50.0 eq, 1.6 mmol) and Et_3N (220 µL, 50.0 eq, 1.58 mmol) were added. The reaction mixture was stirred for 1 hr at room temperature, followed by evaporation by rotary evaporation. Purification by column chromatography (Silica; 0-7% MeOH:DCM) yielded **16** (24.9 mg, 56%). **R$_f$**: 0.18 (Silica; 5% MeOH:DCM). **^{1}H-NMR** (500 MHz; $CDCl_3$): δ 8.02 (t, *J* = 6.9 Hz, 1H), 7.85-7.77 (m, 9H), 7.72-7.60 (m, 9H), 7.46-7.42 (m, 6H), 7.32-7.28 (m, 5H), 7.25-7.13 (m, 5H), 7.05-7.04 (m, 1H), 6.78-6.73 (m, 2H), 6.65-6.62 (m, 2H), 6.56 (t, *J* = 7.3 Hz, 1H), 6.23-6.11 (m, 1H), 4.61-4.56 (m, 1H), 4.47-4.41 (m, 1H), 4.12-4.11 (m, 1H), 3.87-3.85 (m, 2H), 3.73-3.68 (m, 5H), 3.56-3.52 (m, 2H), 3.35-3.22 (m, 6H), 3.09-3.08 (m, 2H), 3.05-3.01 (m, 1H), 2.88 (t, *J* = 6.2 Hz, 2H), 2.80 (d, *J* = 10.6 Hz, 1H), 2.74 (d, *J* = 8.4 Hz, 1H), 1.83-1.76 (m, 1H), 1.62-1.58 (m, 2H), 1.44-1.39 (m, 11H). **HRA-MS(+):** Calculated for $C_{81}H_{81}N_5O_{11}PS^+$ [M^+] 1362.5391; found 1362.5339.

Synthesis of 17. To a solution of **16** (20.1 mg, 1.0 eq, 14.7 µmol) in MeOH (3 mL), I_2 (30.4 mM in MeOH; 0.9 mL, 2.0 eq, 27.4 µmol) was added dropwise over 10 min. Then reaction mixture was quenched with a solution containing 0.2 M sodium citrate and 0.2 M sodium ascorbate at pH ~3-4 until the yellow color disappeared. The quenched reaction mixture was diluted with DCM (20 mL) and washed with brine (20 mL). The aqueous layer was washed again with DCM (15 mL).

The combined organic layers were dried over Na_2SO_4 and evaporated. The crude containing the difulfide product was resuspended in a mixture of 1:1 MeOH-H_2O (3 mL) and TCEP.HCl (40.1 mg, 10.0 eq, 139.9 μmol) was added. The reaction mixture was stirred at room temperature for 30 min, followed by additon of octanoyl anhydride (2 mL) and subsequent additon of Et_3N (60 μL) in portions of 3 μL. The reaction mixture was diluted with DCM (20 mL), and washed with brine (20 mL). The aqueous layer was washed again with DCM (15 mL) and the combined organic layers were dried over Na_2SO_4 and evaporated by rotary evaporation. Purification by column chromatography (Silica; 5-10% MeOH:DCM) yielded **17** (10.4 mg; 56%). Purity was assayed by LC/MS. **R_f**: 0.5 (Silica; 10% MeOH:DCM). **^{1}H-NMR** (500 MHz; $CDCl_3$): δ 8.01 (d, *J* = 7.6 Hz, 1H), 7.84 (dd, *J* = 12.7, 7.7 Hz, 6H), 7.77 (t, *J* = 7.4 Hz, 3H), 7.70-7.59 (m, 8H), 7.16-7.08 (m, 2H), 6.85-6.80 (m, 1H), 6.77-6.75 (m, 1H), 6.64-6.61 (m, 2H), 6.56-6.54 (m, 1H), 4.84-4.52 (m, 3H), 3.97-3.93 (m, 2H), 3.76-3.73 (m, 5H), 3.54-3.47 (m, 3H), 3.34-3.21 (m, 6H), 3.08-3.06 (m, 5H), 2.96 (s, 1H), 2.58-2.30 (m, 2H), 2.36-2.32 (m, 1H), 1.90-1.84 (m, 1H), 1.71-1.58 (m, 4H), 1.52-1.44 (m, 3H), 1.40 (s, 8H), 1.34-1.22 (m, 9H), 0.89-0.81 (m, 3H). **HRA-MS(+)**: Calculated for $C_{70}H_{81}N_5O_{12}PS^+$ [M^+] 1246.5340; found 1246.5264.

Synthesis of mitoDPP-3 (18). A solution of **17** (10.4 mg, 8.02 μmol, 1.0 eq) in 20% TFA:DCM (2 mL) was stirred at room temperature for 30 min, followed by dilution with DCM (10 mL) and evaporation by rotary evaporation. Dilution with DCM (10 mL) and evaporation by rotary evaporation was repeated two more times to remove residual TFA. Purification by columm chromatography (Silica; 10-100% MeOH:DCM) afforded **mitoDPP-3** (**18**; 1.72 mg, 19 %). Purity was assayed by LC/MS. **HRA-MS(+)**: Calculated for $C_{65}H_{74}N_5O_{10}PS^+$ [M^+] 1147.4894; found 1147.4883.

Supplementary References

1 Wieckowski, M. R., Giorgi, C., Lebiedzinska, M., Duszynski, J. & Pinton, P. Isolation of mitochondria-associated membranes and mitochondria from animal tissues and cells. *Nat. Protoc.* **4**, 1582-1590, (2009).

2 Wei-Li, D. *et al.* Functionalized phosphonium-based ionic liquids as efficient catalysts for the synthesis of cyclic carbonate from expoxides and carbon dioxide. *Applied Catalysis A: General* **470**, 183-188, (2014).

3 Dickinson, B. C. & Chang, C. J. A targetable fluorescent probe for imaging hydrogen peroxide in the mitochondria of living cells. *J. Am. Chem. Soc.* **130**, 9638-9639, (2008).

4 Kathayat, R. S., Elvira, P. D. & Dickinson, B. C. A fluorescent probe for cysteine depalmitoylation reveals dynamic APT signaling. *Nat. Chem. Biol.* **130**, 150-152 (2017).
